# Genetic and functional association of *FAM5C *with myocardial infarction

**DOI:** 10.1186/1471-2350-9-33

**Published:** 2008-04-22

**Authors:** Jessica J Connelly, Svati H Shah, Jennifer F Doss, Shera Gadson, Sarah Nelson, David R Crosslin, A Brent Hale, Xuemei Lou, Ty Wang, Carol Haynes, David Seo, David C Crossman, Vincent Mooser, Christopher B Granger, Christopher JH Jones, William E Kraus, Elizabeth R Hauser, Simon G Gregory

**Affiliations:** 1 Department of Medicine and Center for Human Genetics, Duke University Medical Center, Durham, NC, USA; 2 Department of Medicine and Division of Cardiology, Duke University Medical Center, Durham, NC, USA; 3 Miller School of Medicine, University of Miami, Miami, FL, USA; 4 University of Sheffield, Sheffield, UK; 5 GlaxoSmithKline, Philadelphia, PA, USA; 6 University of Wales College of Medicine, Cardiff, UK

## Abstract

**Background:**

We previously identified a 40 Mb region of linkage on chromosome 1q in our early onset coronary artery disease (CAD) genome-wide linkage scan (GENECARD) with modest evidence for linkage (n = 420, LOD 0.95). When the data are stratified by acute coronary syndrome (ACS), this modest maximum in the overall group became a well-defined LOD peak (maximum LOD of 2.17, D1S1589/D1S518). This peak overlaps a recently identified inflammatory biomarker (MCP-1) linkage region from the Framingham Heart Study (maximum LOD of 4.27, D1S1589) and a region of linkage to metabolic syndrome from the IRAS study (maximum LOD of 2.59, D1S1589/D1S518). The overlap of genetic screens in independent data sets provides evidence for the existence of a gene or genes for CAD in this region.

**Methods:**

A peak-wide association screen (457 SNPs) was conducted of a region 1 LOD score down from the peak marker (168–198 Mb) in a linkage peak for acute coronary syndrome (ACS) on chromosome 1, within a family-based early onset coronary artery disease (CAD) sample (GENECARD).

**Results:**

Polymorphisms were identified within the 'family with sequence similarity 5, member C' gene (*FAM5C*) that show genetic linkage to and are associated with myocardial infarction (MI) in GENECARD. The association was confirmed in an independent CAD case-control sample (CATHGEN) and strong association with MI was identified with single nucleotide polymorphisms (SNPs) in the 3' end of *FAM5C*. *FAM5C *genotypes were also correlated with expression of the gene in human aorta. Expression levels of *FAM5C *decreased with increasing passage of proliferating aortic smooth muscle cells (SMC) suggesting a role for this molecule in smooth muscle cell proliferation and senescence.

**Conclusion:**

These data implicate *FAM5C *alleles in the risk of myocardial infarction and suggest further functional studies of *FAM5C *are required to identify the gene's contribution to atherosclerosis.

## Background

Coronary artery disease (CAD), and its extreme manifestation of myocardial infarction (MI), is the leading cause of death in the United States [1] and, concomitant with the epidemic of obesity and diabetes is rapidly becoming the leading cause of death in many developing countries [2]. The genetic predilection of CAD has been well-established; family history has been repeatedly shown to be a independent risk factor even after adjustment for shared environmental factors [3,4]. The heritability of CAD is particularly strong in early-onset forms of the disease (< 50 years of age) where the relative risk of developing early-onset CAD in the first-degree sibling is between 3.8 and 12.1, depending on the age-of-onset in the proband [5].

Myocardial infarction, a serious manifestation of CAD, has also been the subject of genetic analysis. In fact, the only major successes in mapping genes for common, complex cardiovascular disease have focused on the MI phenotype, suggesting that this extreme (but common) manifestation of CAD may be most powerful for disease gene identification. For example, investigators in Iceland carried out a genome wide linkage scan for MI in an Icelandic cohort [6]. Using fine-mapping techniques, the arachidonate 5-lipoxygenase-activating protein (*ALOX5AP*) gene was identified as the major susceptibility gene for MI on chromosome 13q. Although initial results implicated a single gene, expanded analysis of other leukotriene pathway genes identified additional CAD/MI susceptibility genes [7]. Another study of MI revealed evidence for linkage to chromosome 1p34 [8] and later identified apolipoprotein E receptor 2 (*LRP8*) as an underlying susceptibility gene for familial and premature CAD and MI [9]. Most recently, four CAD/MI genome wide association studies (GWAS) [10-13] concomitantly identified genetic association of a region on chromosome 9p21.3 adjacent to the cyclin-dependent kinase inhibitor 2A (*CDKN2A*) and cyclin-dependent kinase inhibitor 2B (*CDKN2B*) genes within independent MI and CAD populations. A study by Broadbent *et al*. validated these findings and identified the antisense noncoding RNA in the *INK4 *locus (*ANRIL*) as the potential disease-causing gene in this region [14]. Despite these successes, these risk variants in combination do not account for the entire underlying genetic component of MI risk, suggesting further studies to identify additional genetic risk factors are necessary.

We previously identified a 40 megabase region of linkage on chromosome 1q25-31 in 420 families from our early onset CAD genome-wide linkage scan (GENECARD) [15], showing modest evidence for linkage (n = 420 families, LOD 0.95). *A priori *stratification by sibling pairs concordant for acute coronary syndrome (ACS; an extreme manifestation of CAD primarily composed of MI) revealed that this modest maximum in the overall group became a well-defined linkage peak (n = 228 families, LOD 2.17, at peak microsatellite marker D1S1589/D1S518). This peak coincides exactly with a quantitative trait locus (QTL) for inflammatory biomarker (monocyte chemoattractant protein 1, MCP-1) linkage in the Framingham Heart Study (LOD 4.27, at peak marker D1S1589) [16], as well as a linkage peak to metabolic syndrome (MetS) from the IRAS family study (LOD 2.59, at peak marker D1S1589/D1S518) [17]. MCP-1 is a potent inflammatory molecule that promotes monocyte/macrophage accumulation in atherosclerotic plaques, which can lead to plaque instability through chronic inflammation and smooth muscle cell proliferation [18]. Metabolic syndrome is a known strong risk factor for development of CAD and MI [19-21]. The overlap of genetic screens in independent data sets provides strong evidence for the existence of a susceptibility gene or genes for CAD in this region.

We conducted a peak-wide association screen of 457 SNPs across the region one LOD score down from the peak marker (168–198 Mb) on chromosome 1q and identified polymorphisms within the 'family with sequence similarity 5, member C' (*FAM5C*) gene that show genetic linkage to and are associated with MI. We confirmed association within an independent CAD case-control dataset (CATHGEN) [22,23] and have identified strong association between SNPs within the 3' end of *FAM5C *and myocardial infarction. We show that *FAM5C *expression is correlated with *FAM5C *genotype in human aortas, suggesting that genetically heritable levels of this molecule in the cells that make up the aorta may play a role in atherosclerosis. Importantly, *FAM5C *is known to promote proliferation, migration, and invasion of pituitary tumors[24], a phenotype relevant to the cellular changes of smooth muscle cells that are associated with the formation and vulnerability of an atherosclerotic plaque [25,26]. We identified *FAM5C *expression in proliferating aortic smooth muscle cells (AoSMCs) and show that levels of this molecule decrease with increasing passage, suggesting that FAM5C levels may play a similar role in SMCs as in pituitary cell tumors. We hypothesize that polymorphisms within this gene may alter *FAM5C *levels in smooth muscle cells, enhancing the atherosclerotic smooth muscle cell phenotype, thus leading to plaque instability and changes in MCP-1 levels.

## Methods

### Early-onset CAD family-based sample (GENECARD)

GENECARD is a collaborative study involving six international investigative sites that make up the GENECARD Study Network. The study is coordinated at Duke University. All study participants signed a consent form approved by the responsible institutional review board or local ethics committee. The study design has been previously reported [27]. Briefly, the initial genome-wide linkage screen within the GENECARD study was composed of 493 affected sibling pairs in 420 families, where at least two siblings met the criteria for early-onset CAD (eoCAD) [15]. In order to obtain a more genetically homogenous sample, using *a priori *defined analyses, our eoCAD sample was stratified into acute coronary syndrome (ACS) families. The presence of ACS was defined as diagnosis of myocardial infarction or unstable angina in at least two affected siblings (228 families) [15]. ACS is a serious manifestation of CAD which includes the subgroup of MI, and is diagnosed by the presence of at least two of the following three signs/symptoms: chest pain typical of CAD, changes on electrocardiogram, and/or positive serum biomarkers for myocardial infarction (MI). Of the 985 affected individuals in the study, 697 met ACS criteria, with 71 individuals with unstable angina only (89% with MI). Given these data, and since MI and ACS represent similar clinical phenotypes, for simplicity we consider the ACS phenotype as primarily a myocardial infarction phenotype. In order to increase the power to detect association in this sample, we included additional individuals from the selected ACS families that were sampled after the initial linkage screen was completed. A total of eight additional parents and 28 additional unaffected siblings were added to the 228 ACS families. Furthermore, two additional families qualified for the ACS phenotype since the original screen and were also included in the analysis. The characteristics of the eoCAD (n = 420) and ACS (n = 230) GENECARD families are summarized in Table 1.

**Table 1:** Clinical characteristics of samples used in this study

Variable	eoCAD Probands	ACS Probands	MI Cases	Controls	Aorta Samples
	(n = 422)	(n = 228)	(n = 368)	(n = 289)	(n = 88)
Age of onset (SD)	44.2 (5.8)	43.9 (6.1)†	51.5 (10.2)†	69.0 (7.0) exam	37.8 (13.2)
Race: Caucasian	92.9%	93%†	100%†	100.0%	87.9%
African American	2.1%	2.2%	0.0%	0.0%	8.6%
American Indian	4.0%	3.0%	0.0%	0.0%	0.0%
Asian	0.7%	0.9%	0.0%	0.0%	2.0%
Hispanic	0.0%	0.0%	0.0%	0.0%	3.5%
Other (unknown)	0.2%	0.4%	0.0%	0.0%	0.0%
Sex: Male	70.6%	72%†	83%†	43.0%	55.2%
Family history of CAD	100.0%	100%†	58%†	28.0%	
Body Mass Index (SD)	29.9 (5.8)	29.9 (6.3)	29.9 (6.8)	28.2 (6.5)	
Smoking	78.0%	84.0%†	72%†	39.0%	
Diabetes	22.3%	22.0%	29.0%	15.0%	
Hypertension	56.9%	57%†	66%†	66.0%	
MI	65.9%*	89.0%*†	100%†	0.0%	Data not available
Sys/Dias BP (SD)	140.1/84.0* (21.5/11.4)	140.6†/87.0* (22.0/12.0)	138.0†/74.0 (24.0/12.0)	149.0/76.0 (23.0/13.0)	
Total Cholesterol (SD)	239.1 (64.8)	235.0† (67.0)	189.0† (56.0)	189.0 (43.0)	
LDL (SD)	146.1 (54.3)	161.0† (125.0)	108† (42.0)	105.0 (35.0)	
HDL (SD)	38.67 (16.3)	40.0 (14.0)	39.0 (12.0)	51.0 (19.0)	
Triglycerides (SD)	230.1 (138.8)	221.0 (157.0)	226.0 (239.0)	169.0 (133.0)	

*significant difference between eoCAD and ACS probands (p ≤ 0.05)

†significant difference between ACS probands and MI cases (p ≤ 0.05)

### MI case-control sample (CATHGEN)

CATHGEN participants were recruited sequentially through the cardiac catheterization laboratories at Duke University Hospital (Durham, North Carolina, United States) with approval from the Duke Institutional Review Board. All participants undergoing catheterization were offered participation in the study and signed informed consent. Medical history and clinical data were collected and stored in the Duke Information System for Cardiovascular Care database maintained at the Duke Clinical Research Institute [28]. Controls and cases were chosen on the basis of extent of CAD as measured by the CAD index (CADi). CADi is a numerical summary of coronary angiographic data that incorporates the extent and anatomical distribution of coronary disease [29]. CADi has been shown to be a better predictor of clinical outcome than extent of CAD [30]. Affected status was determined by the presence of significant CAD defined as a CADi ≥ 32 [31], as we have used for previous studies [22,23]. For patients older than 55 years of age, a higher CADi threshold (CADi ≥ 74) was used to adjust for the higher baseline extent of CAD in this group. Given the linkage to MI in the GENECARD study, we also selected a subgroup of Caucasian individuals who experienced MI from the overall CATHGEN group, similar to the GENECARD ACS families. MI was defined as documentation of MI in the medical history. Controls were defined as ≥60 years of age, with no CAD as demonstrated by coronary angiography and no documented history of cerebrovascular or peripheral vascular disease, myocardial infarction, transplant, or interventional or surgical coronary revascularization procedures [32]. A comparison of clinical characteristics between GENECARD probands and CATHGEN MI cases and unaffected controls is presented in Table 1. As a replication data set, the CATHGEN sample provides 80% power to detect effect sizes between 1.25 and 1.45, for allele frequencies ranging from 0.1 to 0.5. The CATHGEN MI cases were also stratified into a young affected group (CAD AOO ≤ 55), which provides a consistent comparison for the GENECARD family study.

### Human donor aorta samples and expression

Human aorta samples were collected from heart transplant donors as previously described [33]. DNA and RNA were isolated from each sample. Eighty-eight samples in total were analyzed representing fifty-eight unique samples, as 30 individuals had more than one sample. As harvested tissue was obtained from deceased heart donors, the clinical data associated with these aortas is very limited and consists of age, sex, and race (Table 1). Genotyping was performed using Applied Biosystems Taqman allelic discrimination assays and expression profiling was performed using Affymetrix GeneChip U95Av2 (Affymetrix, Santa Clara, CA). Expression signal intensity values were log_2 _transformed and normalized using quantile normalization. We analyzed cis effects of *FAM5C *variants on *FAM5C *expression using the Affymetrix tag 34442_at, representing the 3' end of *FAM5C*. Because some subjects had multiple samples while others did not, we treated each individual and sample separately. The expression level of the tag was modeled using multiple linear regression including age, sex, race and additive genotype. To account for repeated measures (i.e., multiple sections per subjects) and as a validation, a mixed model as implemented in the SAS PROC MIXED procedure was utilized (SAS Institute Inc., Cary, NC).

### Peak-wide SNP selection

The approximate 30 Mb region of linkage on chromosome 1q contains 138 genes (NCBI Build 36 in Ensembl). We used an iterative fine mapping approach that involved selecting SNPs with a minor allele frequency of at least 5% in the Caucasian population spaced at 100 kilobases (kb) [34]. These SNPs were intercalated with at least one previously identified and confirmed (validated) SNP to represent positional candidate genes from NCBI Build 35 (120 genes). When possible, coding SNPs, SNPs that altered a splice site or that were contained within putative regulatory regions of multi-species sequence conservation (chimp, mouse, rat, dog, chicken, Fugu, zebrafish) were selected for genotyping. If the gene was greater than 100 kb in size, multiple SNPs were selected using the same criteria. Additionally, SNPs were selected from regions that do not contain annotated genes at an average SNP to SNP distance of 100 kb, while taking into account regions of multi-species sequence conservation.

Using these criteria, 231 intragenic and 226 intergenic SNPs were selected for genotype analysis within the chromosome 1q linkage region with an average spacing of 66 kb. Twenty-seven genes contained validated coding SNPs with allele frequencies >5%; twenty-two genes contained synonymous SNPs with allele frequencies >5%; eleven SNPs are found within untranslated regions (5' or 3' UTR); and one hundred and seventy-one intronic SNPs were selected, the reference allele is conserved within fifty-five of these (Additional files, Table 1).

### SNP genotyping

A total of 457 SNPs were genotyped at two genotyping facilities. 384 SNPs were genotyped at the University of Washington through a grant from SeattleSNPs (see Availability and requirements section for URL). Genotyping in the GENECARD sample was performed by SeattleSNPs using the Illumina BeadStation 500 G SNP genotyping system (Illumina, San Diego, California, United States). Each Sentrix Array generates 384 genotypes for 96 individuals; within each individual array experiment, four samples purchased from Coriell were included as quality control samples. The Coriell samples were of Centre d'Etude du Polymorphisme Humain (CEPH) (Caucasian) origin, Asian and Hispanic ethnicity and were used for gender checking and to assess sample quality. In addition, one sample per plate was duplicated for internal quality control. These samples were used to identify possible sample plating errors and genotype calling inconsistencies. Twenty-two SNPs did not cluster well and were not called. The call rate for the remaining 362 SNPs was 99.8%. One replicate error was identified in SNP rs2370025, all other SNPs yielded greater than 99.999% concordance among the replicates.

Ninety-five additional inter- and intragenic SNPs were assayed at the Center for Human Genetics using Taqman allelic discrimination assays. A total of 15 quality control samples – composed of six reference genotype controls in duplicate, two CEPH pedigree individuals, and one no-template sample – were included in each quadrant of the 384-well plate. SNPs that showed mismatches on quality-control samples were reviewed by an independent genotyping supervisor for potential genotyping errors. All SNPs examined were successfully genotyped for 95% or more of the individuals in the study. Error rate estimates for SNPs meeting the quality control benchmarks were determined to be less than 0.2%.

### Statistical analysis

All SNPs were tested for deviations (p ≤ 0.0001) from Hardy-Weinberg equilibrium (HWE) in the affected and unaffected race-stratified groups. Two SNPs deviated from HWE in the peak-wide association study, rs1503122 (cases) and rs438781 (cases and controls). Neither of these SNPs was statistically significant in our association analysis. Interestingly, rs438781 lies within a reported copy number polymorphism, which is most likely the cause of the disequilibrium. In CATHGEN, one SNP, rs9427746, significantly deviated from HWE (p < 0.0001) in the Caucasian control group. This deviation was neither due to mixed racial backgrounds as all of the samples were from Caucasian origin, nor was it due to assay dropout as all of our controls are randomly interspersed with our cases in the 384 well plates. This deviation is either a significant departure representing an association or due to random chance. Linkage disequilibrium between pairs of SNPs was assessed using the Graphical Overview of Linkage Disequilibrium package [35] and displayed using Haploview [36].

In the initial screen, two-point linkage using a dominant and recessive model was performed using Fastlink [37,38] and Homog [39] and family-based association was tested using the Association in the Presence of Linkage (APL) test [40]. We analyzed the GENECARD families together regardless of race because these families formed the basis of the original linkage evidence. We chose to use the APL test for family based association because this test incorporates data from affected sibling pairs with available parental data and unaffected siblings in the analyses, effectively using all available information in the GENECARD families. The APL software infers missing parental genotypes, appropriately accounts for the non-independence of affected siblings and calculates a robust estimate of the variance. APL results from markers with variance estimates of less than five are viewed as less reliable [41]. A single SNP in *TROVE2 *was significant in our original screen with a variance less than five and did not replicate.

In addition to APL, family-based association of *FAM5C *SNPs was performed using the Pedigree Disequilibrium Test (PDT) [42,43]. We employed this additional test at the gene level in order to obtain the most information about each polymorphism. The PDT test uses related trios and discordant sibships from extended pedigrees and like other family-based association tests is robust to population stratification. In order to determine if replicated SNPs could account for the MI linkage signal we employed Linkage and Association Modeling in Pedigrees (LAMP). LAMP estimates the degree of linkage disequilibrium (LD) between a candidate SNP and the putative disease locus through joint modeling of linkage and association [44,45].

Allelic association in the CATHGEN MI cases and controls was evaluated using multivariable logistic regression modeling adjusted for sex, and known CAD risk factors (history of hypertension, history of diabetes mellitus, body mass index, history of dyslipidemia, and smoking history) as covariates. These adjustments could hypothetically allow us to control for competing genetic pathways that are independent risk factors for CAD, thereby allowing us to detect a separate CAD genetic effect. SAS 9.1 (SAS Institute, Cary, North Carolina, United States) was used for statistical analysis.

### FAM5C sequencing

**Figure 1:**
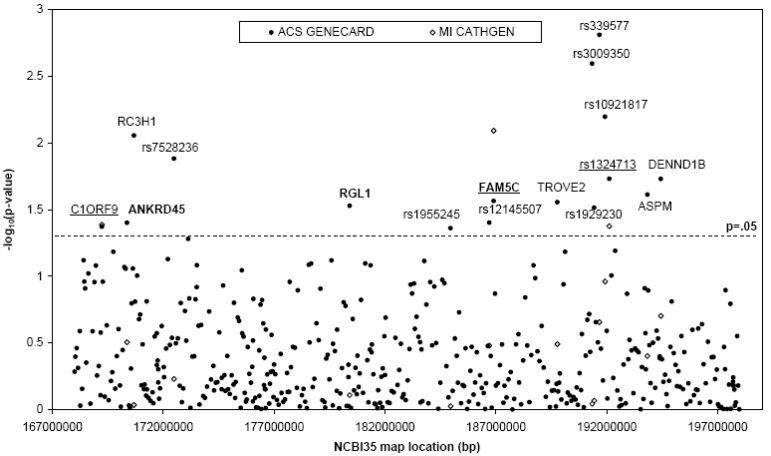
**Chromosome 1 peak-wide mapping of genes involved in myocardial infarction**. 457 SNPs were genotyped across the 1 LOD down region of linkage on chromosome 1 in the GENECARD ACS sample. Association was assessed using APL. The -log_10 _of the p-value is plotted according to chromosome position (closed circles). Markers with a p-value less than 0.05 fall above the dashed line; labels identify these genes and intergenic SNPs. Markers in bold exhibited modest evidence for linkage (LOD ≥ 1) in two point analysis. Sixteen markers that were associated (p ≤ 0.05) in the GENECARD ACS sample were genotyped in a MI case-control population (open circles). Markers which show replication are underlined.

To identify novel SNPs within seven regions of multi-species sequence conservation localized to the last intron of *FAM5C *(see Additional files, Figure 1, arrows), PCR amplicons were generated using standard conditions and sequenced using ABI Big Dye v3.1 and an ABI 3730 automated sequencer (primers available upon request). Sequencing data were analyzed using Sequencher software (GeneCodes, Ann Arbor, Michigan, United States). All amplicons were generated within 16 affected and 16 unaffected GENECARD individuals (16 Caucasian males and 16 Caucasian females). Primer sets for each region are listed in Additional files, Table 2.

**Table 2:** LAMP Analysis in GENECARD ACS families and replication in CATHGEN MI cases and controls

SNP	LOCATION (bp)	Alleles		LINKAGE TEST (df = 3)		ASSOCIATION TEST (df = 1)		OTHER LINKED VARIANTS (df = 2)		CATHGEN MI Logistic regression†		CATHGEN Minor Allele Frequency
		Major	Minor	LOD	pvalue	LOD	pvalue	LOD	pvalue	Allele pvalue	Allele OR (95% CI)*	
RS7514392	188,323,178	C	g	2.95	0.004	0.33	0.220	1.69	0.020	-	-	-
RS10920501	188,328,568	A	t	2.98	0.003	0.96	**0.040**	2.27	0.005	**0.018**	0.612 (0.408–0.919)	0.18
RS12142564	188,338,626	C	t	2.80	0.005	1.43	**0.010**	2.53	0.003	0.322	0.799 (0.508–1.257)	0.11
RS2185836	188,349,217	A	g	2.89	0.004	0.76	0.060	2.82	0.002	0.273	0.791 (0.521–1.202)	0.45
RS2419370	188,375,504	T	c	2.50	0.009	0.56	0.110	2.49	0.003	**0.003**	1.925 (1.251–2.961)	0.44
RS2990996	188,411,850	C	a	3.19	0.002	0.16	0.400	3.18	0.001	0.115	1.441 (0.915–2.271)	0.50
RS815343	188,422,256	C	t	2.97	0.003	0.34	0.210	2.31	0.005	**0.005**	0.574 (0.392–0.842)	0.39
RS1891586	188,430,617	T	g	2.95	0.004	0.04	0.700	2.93	0.001	**0.003**	0.563 (0.385–0.823)	0.23
RS9427746	188,438,263	G	c	2.86	0.004	0.01	0.810	2.50	0.003	**0.007**	0.587 (0.399–0.863)	0.40
RS10920678	188,506,530	G	a	2.99	0.003	0.01	0.850	2.77	0.002	0.159	0.753 (0.508–1.117)	0.43
RS480692	188,526,284	T	c	3.29	0.002	0.41	0.200	3.42	0.000	**0.028**	1.591 (1.052–2.405)	0.43
RS12724000	188,618,300	C	t	2.78	0.005	0.04	0.660	2.60	0.003	**0.031**	0.645 (0.434–0.961)	0.17
RS872177	188,659,068	T	a	2.93	0.004	0.05	0.600	2.90	0.001	0.188	0.776 (0.533–1.132)	0.31
RS2134098	188,660,894	G	c	2.82	0.005	0.89	**0.040**	3.00	0.001	0.131	0.679 (0.411–1.122)	0.08
RS10920722	188,694,717	A	t	2.65	0.007	0.07	0.600	2.37	0.004	**0.009**	0.589 (0.397–0.874)	0.19
RS10920725	188,707,160	T	c	2.65	0.007	0.13	0.400	2.45	0.004	**0.007**	0.584 (0.394–0.865)	0.20
RS11581737	188,726,896	G	a	2.87	0.004	0.18	0.400	2.76	0.002	**0.031**	0.642 (0.429–0.960)	0.16

*Odds Ratio (OR) calculated for minor allele, - not tested

†adjusted for sex, history of hypertension, history of diabetes mellitus, body mass index, history of dyslipidemia, and smoking history

### Aortic endothelial and smooth muscle cell culture

Cryopreserved human aortic smooth muscle (AoSMC) and human aortic endothelial cells (HAEC) were obtained from Lonza (Walkersville, MD) at passage 3 and cultured using SGM-2 or EGM-2 media (Lonza) until the indicated passage. Cells were grown in T-75 SoLo Flasks Nunclon™Δ (NUNC, Rochester, NY) according to manufacturer's instructions (see Availability and requirements section for URL).

### Reverse transcriptase PCR (RT-PCR)

RNA from 20 human tissues (Human Total RNA Master Panel II) was obtained from Clontech (Mountain View, CA). Lot number 6020107 contained RNA from the following tissue types: Fetal Brain, Spinal Cord, Skeletal Muscle, Lung, Trachea, Fetal Liver, Liver, Bone Marrow, Kidney, Thymus, Thyroid, Adrenal Gland, Salivary Gland, Uterus, Testes, Prostate, and Placenta. RNA from cultured aortic smooth muscle and endothelial cells was isolated using a Ribopure Kit from Applied Biosystems (Foster City, CA). cDNA was synthesized using the protocol for cDNA synthesis in the Illumina Total Prep RNA Amplification kit from Applied Biosystems (Foster City, CA). Primers corresponding to exon 6 and exon 7 of *FAM5C *were used for PCR from cDNA (5'-ACAACAGTGACTTTGAGGAATCAGA-3' and 5'-GCGCTGAAAATTAGAATCCATTG-3'). Primers corresponding to exons 8 and 9 of *GAPDH *were used as a template control (5'-CTCCTCCACCTTTGACGCTG-3', and 5'-AGGGGAGATTCAGTGTGGTG-3'). 20 ng of cDNA template was used for each reaction and Platinum Taq polymerase (Invitrogen, Carlsbad, CA) was used for all PCR reactions. PCR reaction conditions were as follows: Step 1: 95°C for 5 minutes, Step 2: 30 cycles (*FAM5C*) or 20 cycles (GAPDH) of 95°C for 1 minute followed by 62°C for 1 minute, and 72°C for 1 min, and Step 3, 72°C 10 min.

### FAM5C real time RT-PCR

Applied Biosystems Taqman Gene expression assays were used to perform quantitative (real time) RT-PCR (FAM5C, Hs00982332_m1 and GAPDH, Hs02758991_g1). The following reaction components were used for each probe: 2 uL cDNA, 5 ul Custom TaqMan SNP Master Mix (Applied Biosystems, Foster City, CA), 0.5 ul of assay, and 2.5 ul water. Reactions were performed in a single 384 well plate in triplicate using an ABI PRISM^® ^7900 HT Sequence Detection System. PCR reaction conditions were as follows: Step 1: 50°C for 2 minutes, Step 2: 95°C for 10 minutes, Step 3: 40 cycles of 95°C for 15 seconds followed by 60°C for 1 minute. Expression relative to *GAPDH *was calculated using 2^ΔCt ^[46] and levels were normalized to heart expression.

## Results

### Peak-wide genotyping and analysis in GENECARD ACS linkage Peak

457 SNPs, at an average spacing of 66 kilobases, were genotyped in the one LOD down support interval (169–199 Mb) of the GENECARD chromosome 1q ACS linkage peak (see Additional files, Table 1). Family-based association performed in the ACS families using APL identified sixteen SNPs that were significant at p ≤ 0.05 (Figure 1, Additional files, Table 3), three of which also displayed linkage: rs10912660 in the ankyrin repeat domain 45 gene (*ANKRD45*), rs12092963 in the ral guanine nucleotide dissociation gene (*RGL1*), and rs1891586 in *FAM5C *(Figure 1, bold). The clinical characteristics comparing the ACS probands in GENECARD to the MI cases in CATHGEN show the suitability of this group for replication (Table 1). Three of the sixteen SNPs replicated in the CATHGEN MI cohort at p ≤ 0.05 (Figure 1, underlined). Two SNPs, rs2239816 and rs1891586, reside within genes, chromosome 1 open reading frame 9 protein (*C1orf9*) and *FAM5C*, and the third within an intergenic region (rs1324713).

A SNP found within the first intron of the tumor necrosis factor superfamily 4 gene (*TNFSF4*), rs3850641, residing within the chromosome 1q linkage peak, has previously shown to be associated with MI in two independent populations [47], but although it was linked, was not associated in the GENECARD samples (Additional files, Table 4). LAMP analysis determined that this SNP does not account for the linkage observed in these families (data not shown).

### tagSNP genotyping and analysis

We chose haplotype tagging SNPs in *C1orf9*, *FAM5C *and the intergenic region 100 kilobases upstream and downstream of rs1324713. None of the additional genotyped tagSNPs in *C1orf9 *or the intergenic region displayed both association and linkage (Additional files, Table 4). However, the original *C1orf9 *SNP identified in the screen, rs2239816, is significantly associated in both GENECARD ACS (APL, p = 0.05) and CATHGEN MI samples (allele p = 0.04) and results in an amino acid change at position 11 (Pro11Ser). This SNP does not, however, explain the linkage seen in our ACS family subset.

**Figure 2:**
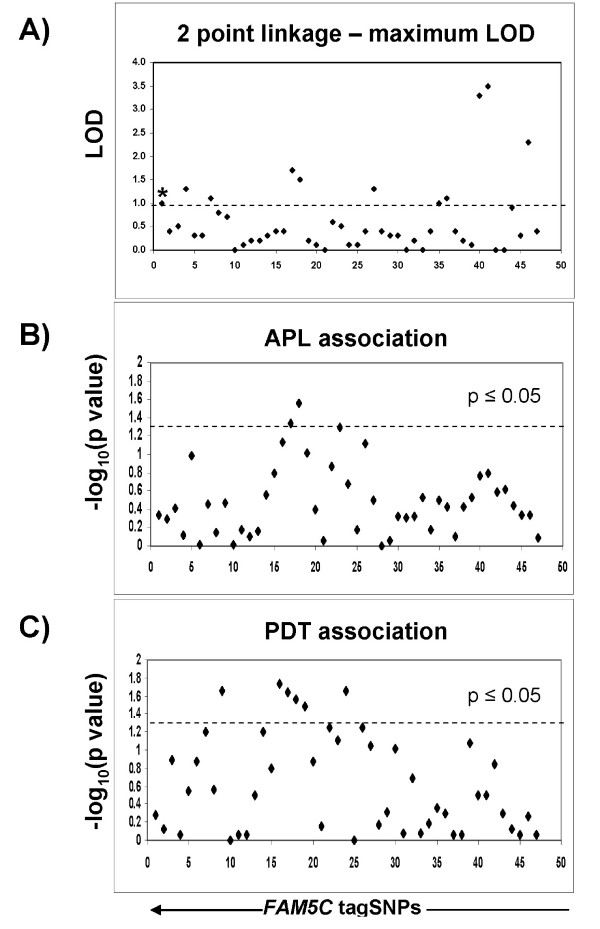
***FAM5C *tagSNPs are associated and linked to acute coronary syndrome**. 47 haplotype tagging SNPs were selected to span the length of FAM5C as well as 15 kb up and 5 kb downstream of the gene to account for potential regulatory elements. Parametric linkage (A), APL association (B) and PDT association (C) were assessed. SNPs that were linked (LOD ≥ 1) and/or associated (p ≤ 0.05) were selected for replication (markers that fall above the dashed lines, n = 16). * denotes rs10920501.

**Figure 3:**
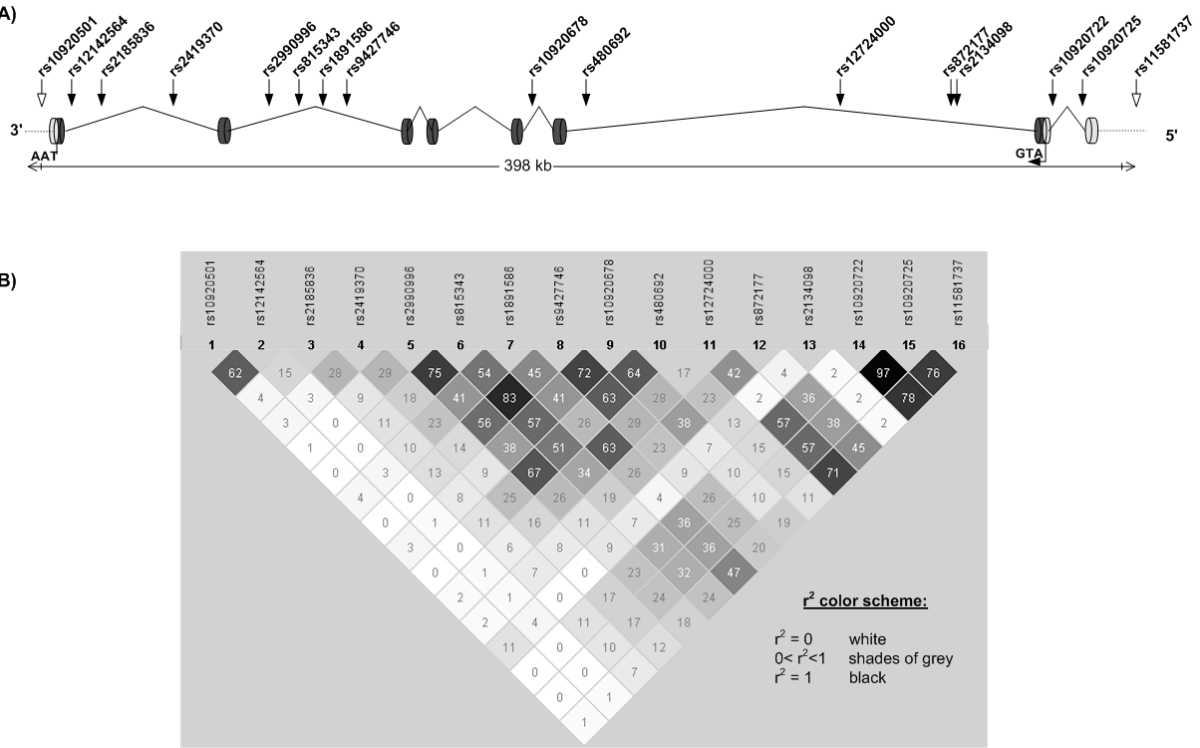
**Schematic of *FAM5C *gene structure and pairwise LD between *FAM5C *SNPs**. (A) Sixteen of forty-seven tagSNPs in FAM5C that were tested for replication and linkage are shown. SNPs with an open arrow are found outside of the gene. All sixteen SNPs represent 16 of 47 linkage disequilibrium (LD) blocks. (B) LD was estimated in one unaffected Caucasian individual from each non-redundant GENECARD ACS sibling pair. A similar pattern of LD was observed using the CATHGEN MI affected and unaffected individuals.

A single SNP in *FAM5C *(rs1891586) from the peak-wide screen showed both linkage and association in the ACS sample (maximum LOD = 1.54, p = 0.027). To further fine map the gene we selected 67 haplotype tagging SNPs using HapMap data and Tagger [48] (capturing 100% of the alleles at r^2 ^= 0.8 in the European population) from a region that spanned *FAM5C *and 15 kilobases upstream and 5 kilobases downstream of the gene. Only 48 of these SNPs had minor allele frequencies >5% and were informative given the small sample size when genotyped in the GENECARD ACS families. Sixteen of these SNPs displayed linkage (LOD ≥ 1 in either model) and/or association (p ≤ 0.05) (Figure 2, Figure 3, Additional files, Table 5), with three SNPs (rs2134098, rs12142564 and rs10920501) partially accounting for the linkage signal on chromosome 1 (Table 2, compare association test to other linked variants) by LAMP analysis [44,45]. In the CATHGEN sample, twelve of these sixteen SNPs were significantly associated with MI (Table 2), however, only one of the three SNPs that partially accounted for linkage in GENECARD (rs10920501) was associated with MI in CATHGEN. Interestingly, when we stratified our MI sample by age of onset (see methods), we found that the strength of the overall rs10920501 signal increased within the young affected group (T allele, p = 0.009, OR = 0.539). This SNP tags (r^2 ^≥ 0.8) 6 additional SNPs that flank the last exon on *FAM5C *(see Addition files, Figure 1). This SNP is in linkage equilibrium (r^2 ^= 0.04) with rs1891586, the original SNP identified in the peakwide screen.

### Sequencing FAM5C conserved regions in intron 7

We noted blocks of multi-species sequence conservation in the last intron of *FAM5C *and hypothesized that the causal SNP, in LD with rs10920501, may lie in one of these regions. The allele frequency of rs10920501 is approximately 16% in the Caucasian population. We rationalized that a causal SNP that was in LD with rs10920501 would be at a similar allele frequency. Given Kruglyak and Nickerson's estimates [49] we performed sequencing across 32 individuals that should allow for a SNP detection rate between 99% and 99.9% at a minor allele frequency of 10%. The seven conserved regions (see Additional files, Figure 1, arrows) were PCR amplified from 16 Caucasian cases and 16 Caucasian controls using standard conditions and sequenced. No novel SNPs were identified (data not shown).

### FAM5C allele specific gene expression

**Figure 4:**
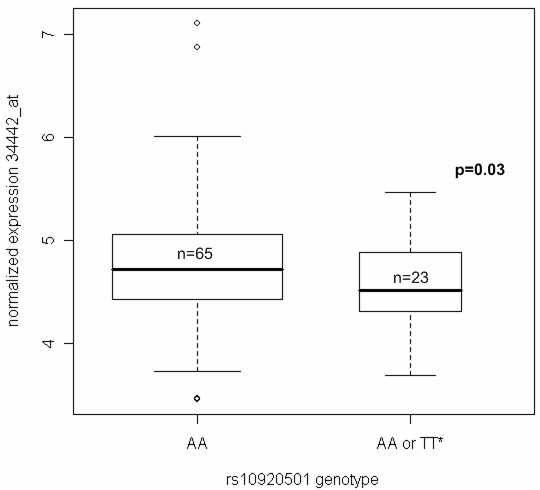
**FAM5C expression is modulated by the T allele of rs10920501**. Eighty-eight human donor aorta were harvested, genotyped for rs10920501, and assayed for FAM5C expression using Affy tag 34442_at. Box plots are shown for gene expression by genotype. The presence of the T allele of rs10920501 significantly reduces FAM5C expression. The p-value shown is for the generalized model adjusted for age, sex, and race. * n = 1 in the TT genotype group.

We next asked if rs10920501 (or an unidentified SNP in linkage disequilibrium with rs10920501) was functionally relevant to CAD. We genotyped a set of aorta samples that we had previously collected for expression analysis [33] and we found that the inheritance of the T allele of rs10920501 is significantly associated with a reduction in the expression of *FAM5C *in aorta (Figure 4) (p = 0.03 in generalized model; p = 0.02 in mixed regression model to account for repeated measures). Since the T allele is protective in our sample (allelic odds ratio, 0.609 in CATHGEN, Table 2) and carriers of the T allele show decreased FAM5C gene expression in aorta, higher expression of *FAM5C *in the cells that make up the artery may be related to increased risk of MI. Because a QTL for MCP-1 in the Framingham Heart Study overlaps with the GENECARD ACS linkage peak, we also tested the effect of rs10920501 on *MCP-1 *expression levels in the aorta, but found no evidence for any relationship (data not shown).

### FAM5C is expressed in proliferating human aortic smooth muscle cells and expression changes with passage

**Figure 5:**
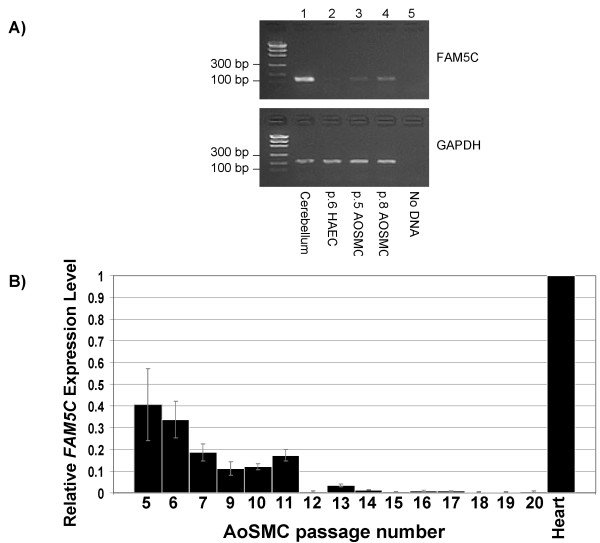
***FAM5C *is expressed in aortic endothelial and smooth muscle cells**. (A) RT-PCR was performed on RNA derived from the cerebellum (control) and proliferating aortic endothelial (HAEC, passage 6) and smooth muscle cells (AoSMC, passage 5 and 8). (B) Real time RT-PCR was performed on RNA isolated from serially passed AoSMC. Aliquots were taken at every passage and experiments were performed in triplicate. *FAM5C *and *GAPDH *expression were assayed at the same time and *GAPDH *was used to normalize all samples. The expression level was calculated relative to heart RNA (control). Standard deviation was calculated for each experiment and is indicated by grey bars.

Given that *FAM5C *is expressed in human aorta, we sought to determine if *FAM5C *is expressed in smooth muscle and endothelial cells, the primary constituents of the human aorta. We cultured proliferating aortic smooth muscle (AoSMC) and human aortic endothelial cells (HAEC) and isolated RNA. Reverse transcriptase PCR (RT-PCR) identified *FAM5C *expression in both endothelial and aortic smooth muscle cells (Figure 5A, lanes 2, 3, and 4). In order to determine if *FAM5C *expression was specific to the brain and arteries, we assayed expression of *FAM5C *in 20 human tissues and found that it is expressed in many different tissue types at varying levels (Additional files, Figure 2).

We noted that the expression levels of *FAM5C *appeared to vary with AoSMC passage (compare p.5 and p.8 AoSMC RT-PCR Figure 5 and Additional files, Figure 2) and therefore hypothesized that *FAM5C *levels may change with proliferative capacity and cellular senescence. Sub-culturing of primary cells, such as AoSMC, results in a progressive reduction of proliferation until the cells reach senescence, described as Hayflick's limit [50]. We serially cultured AoSMC derived from a 29 year old Caucasian male donor until senescence (passage 20) indicated by cessation of proliferation and an enlarged flattened cell morphology. We measured the level of *FAM5C *expression in triplicate during each passage (passage 8 RNA was depleted) using real time RT-PCR. We found that *FAM5C *levels decrease as cells age in culture (Figure 5B).

## Discussion and Conclusion

We herein report the results of fine-mapping a linkage peak for MI and identifying a novel MI susceptibility gene, *FAM5C*. We show that polymorphisms in *FAM5C *are associated with MI in two independent samples and that the SNP, which partially accounts for the linkage in our GENECARD ACS peak, is also significantly associated with changes in *FAM5C *gene expression in the human aorta. To begin to address the functional role of FAM5C in MI, we observed that *FAM5C *is expressed in the human aorta and that its transcript levels decrease with increasing passage of AoSMC, suggesting that the level of gene expression may play a role in proliferation and senescence of this cell type.

*FAM5C *was originally identified in the mouse brain as a gene that is induced by bone morphogenic protein and retinoic acid signaling (*BRINP3 *gene) [51] and has recently been shown to be over-expressed in human pituitary tumors [24]. In the aforementioned study, the authors show that FAM5C is localized to the mitochondria and that over-expression of this molecule leads to increased proliferation, migration, and invasion of non-tumorogenic pituitary cells (the opposite being true for knockdown of *FAM5C*). Through complex signaling cascades, mitochondria have the ability to activate multiple pathways that modulate both cell proliferation and, conversely, promote cell arrest and programmed cell death (reviewed in [52,53]). FAM5C localization to the mitochondria and its putative role in regulating cell proliferation and migration provide an intriguing hypothesis for the role of *FAM5C *in smooth muscle cells and the formation and vulnerability of the atherosclerotic plaque. SMCs move from a contractile state to a proliferative, migratory state in the presence of endothelial cell dysfunction at the initiation phase of plaque formation. They migrate into the region of plaque formation and form the fibrous cap that covers the plaque which is prone to rupture (reviewed in [25]). *FAM5C *levels could play a role in the initiation of smooth muscle cell proliferation and migration and/or in the disintegration of the smooth muscle cell in the fibrous cap. We are currently conducting experiments to determine if *FAM5C *levels have effects on smooth muscle cell proliferation, migration, apoptosis and senescence.

While our analysis was primarily aimed at an MI phenotype, our ACS family sample included a limited number of individuals with unstable angina. As a result the power to detect a distinct MI effect may be somewhat lower in our ACS family data set. Nevertheless, we observed consistent and replicated results in the MI case-control set and in the ACS family data set, suggesting that our results are robust to potential phenotypic heterogeneity. In addition, two recent WGA studies from the Framingham Heart Study have identified significant association between atrial fibrillation (AF) and *FAM5C *(rs1604355), and also for left atrial size (LAS) and *FAM5C *(rs1935881) [54,55]. Both of these SNPs are in low LD (r2 < 0.1) with rs10920501; however, a SNP within the linkage disequilibrium bin containing rs10920501 was not represented on the SNP chip used in these studies, leaving open the possibility that rs10920501 may also play a role in these phenotypes. Although atrial fibrillation and left atrial size are very different phenotypes from MI, these data, together with the data presented in this paper, suggest that further genetic and functional studies are warranted to determine the role of *FAM5C *and variation within this gene in diverse diseases in cardiovascular tissues.

Three separate studies identified linkage to the same region of chromosome 1, the Framingham Heart Study [16], the IRAS family study [17], and the GENECARD study [15]. Through careful fine mapping of a linkage peak for an extreme CAD phenotype, MI, we have defined a single gene that can partially account for the linkage in this peak. The relationship between MCP-1 levels, metabolic syndrome, MI, and FAM5C still remains to be defined. Inflammation lies at the heart of these pathophysiologies. We know that inflammation is an important part of metabolic syndrome [56], individuals with metabolic syndrome are at higher coronary disease risk [57], and metabolic syndrome increases the risk of an event (MI, revascularization, or cardiac death) in individuals with a family history of MI [58]. In addition, vascular SMC pathophysiology is related to inflammation which could explain the MCP-1 QTL linkage. Further work is clearly indicated in this area.

One of the more difficult aspects of genetic association analysis is how to appropriately correct for multiple comparisons in evaluating the statistical significance of any given result. Possible approaches range from the most conservative Bonferroni correction, to estimation of false discovery rate [59], to weighted corrections of combined data, to no correction at all. We are aware that the p-values presented in this paper were not corrected for multiple comparisons. Our enthusiasm for *FAM5C *as an MI susceptibility gene is based on the observed replication of association results from multiple independent data sets and different approaches. The line of reasoning has several parts. First, rs10920501 represents a significant marker that can partially account for the linkage signal in our ACS families, and the statistical significance of the observation replicates in an independent case-control sample. Second, the probability of observing three or more significant results out of sixteen in the independent case-control sample under the null hypothesis that there is no association (p ≤ 0.05) is 0.043. Third, we have observed that the level of the transcript in the aorta is associated with genetic variation this SNP (or a SNP LD with it). Last, we observed that levels of *FAM5C *change with passage in AoSMC, implying that genetic variation in the gene might be involved in AoSMC cellular processes involved in the formation of unstable atherosclerotic plaque leading to MI. Further work to elucidate the function of *FAM5C *in MI, including the MI-associated polymorphisms and its downstream targets, is necessary in order to begin to understand the role this mitochondrial molecule plays in this disease.

## Competing interests

The authors declare that they have no competing interests.

## Availability and requirements

SeattleSNPs: 

Lonza: 

## Authors' contributions

JJC, JFD, and SGG carried out the molecular genetic studies. JJC and JFD carried out the RTPCR and real time RTPCR. ABH, DRC, XL, SN and CH performed the statistical analysis. TW performed bioinformatic analyses. DS provided expression data from human aorta. DCC, VM, CBG, CJHJ, WEK provided the clinical data and samples for genotyping. JJC, SHS, ERH and SGG conceived of the study, and participated in its design and coordination and helped to draft the manuscript. All authors read and approved the final manuscript.

## Pre-publication history

The pre-publication history for this paper can be accessed here:



## Supplementary Material

Additional file 1 **Figure 1**. **The location of rs10920501 and the SNPs it represents**. UCSC genome browser output for chr1:188,266,509–188,418,688 basepairs (build 36) containing the 3' end of the *FAM5C *gene. The sequenced regions of high conservation present in the last intron of *FAM5C *are indicated by black arrows. Click here for file

Additional file 2 **Figure 2**. ***FAM5C *is ubiquitously expressed in human tissues**. *FAM5C *RT-PCR was performed on RNA derived from 20 human tissues (as labeled) and proliferating aortic endothelial (HAEC, passage 6) and smooth muscle cells (AoSMC, passage5 and 8). *GAPDH *is displayed to assess template input. Click here for file

Additional file 3 **Table 1**. The list of SNPs genotyped in the peakwide screen. Click here for file

Additional file 4 **Table 2**. A list of primer sets used to amplify genomic regions of *FAM5C *for sequencing. Click here for file

Additional file 5 **Table 3**. Sixteen significant SNPs identified in peakwide screen of chromosome 1 ACS linkage peak and replication of three associated SNPs in CATHGEN MI case-control sample. Click here for file

Additional file 6 **Table 4**. tagSNP genotyping results for *C1orf9 *and an intergenic region surrounding rs1324713 in GENECARD ACS families. Click here for file

Additional file 7 **Table 5**. tagSNP genotyping results for *FAM5C *in GENECARD ACS families. Click here for file
