# Supplementary figures and images for: Genetic and functional association of FAM5C with myocardial infarction
Source: BMC Med Genet. 2008 Apr 22;9:33. doi: 10.1186/1471-2350-9-33 (PMC2383879; doi:10.1186/1471-2350-9-33)

# chr1:188,266,509-188,418,688

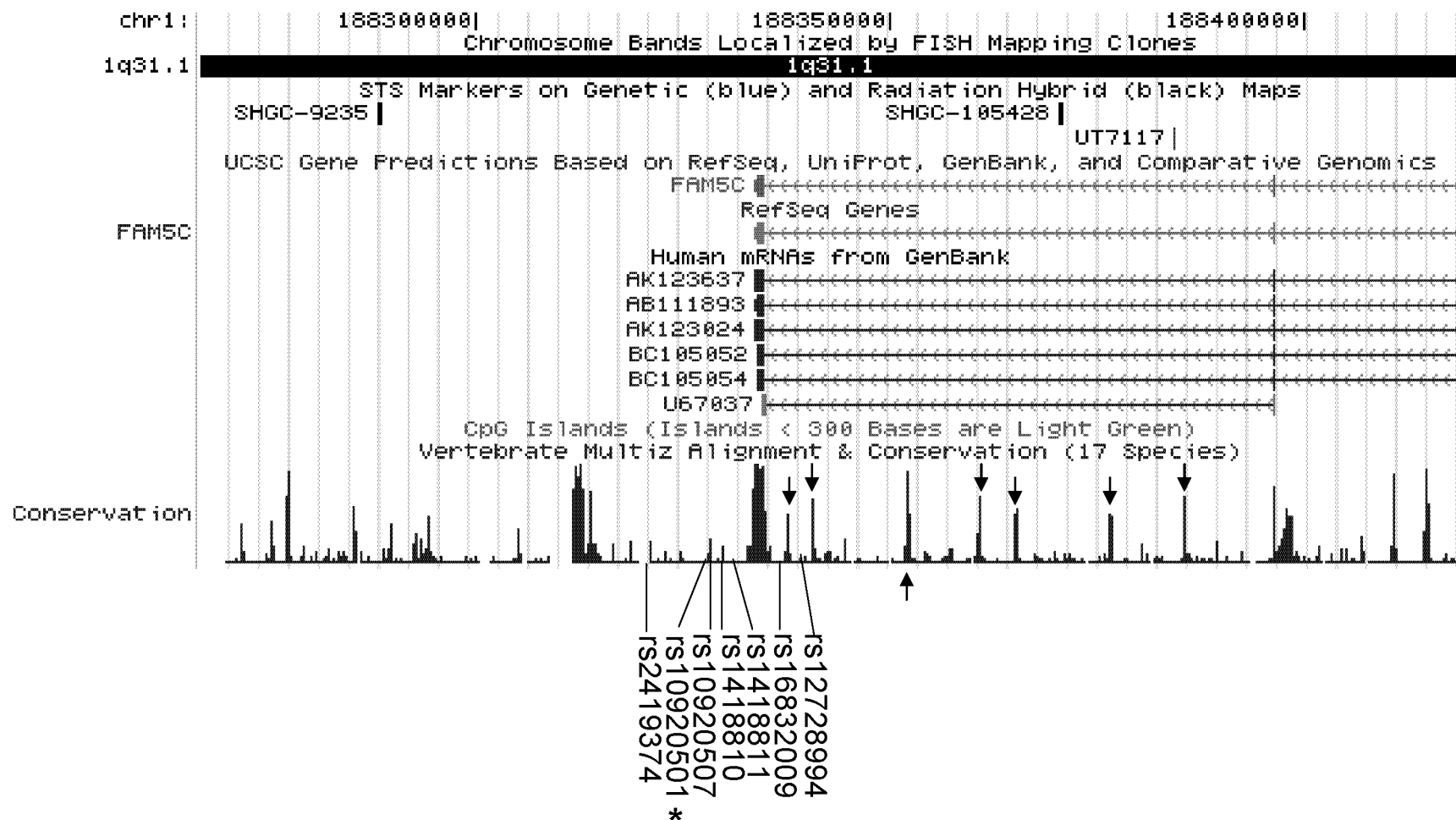

Supplement: Additional file 1 — Figure 1. The location of rs10920501 and the SNPs it represents. UCSC genome browser output for chr1:188,266,509–188,418,688 basepairs (build 36) containing the 3' end of the FAM5C gene. The sequenced regions of high conservation present in the last intron of FAM5C are indicated by black arrows. [file 1471-2350-9-33-S1.pdf]
