# Supplementary material for: Genetic and functional association of FAM5C with myocardial infarction
Source: BMC Med Genet. 2008 Apr 22;9:33. doi: 10.1186/1471-2350-9-33 (PMC2383879; doi:10.1186/1471-2350-9-33)
Supplement: Additional file 3 — Table 1. The list of SNPs genotyped in the peakwide screen. [file 1471-2350-9-33-S3.doc]

| Additional files, Table 1 | | | | |
| --- | --- | --- | --- | --- |
| dbSNP | Gene Symbol | Reference Allele Frequency | NCBI Build 36 Location | SNP Type |
| RS2224783 | *DNM3* | 0.58 (A) | chr. 1 168900685 | Intron, Transition Substitution |
| RS12070766 |  | 0.85 (A) | chr. 1 169539124 | Intergenic/Unknown,Transition Substitution |
| RS2223477 | *FMO4,TOP1P1* | 0.39 (G) | chr. 1 169575456 | Intron,Transition Substitution |
| RS11588367 |  | 0.16 (G) | chr. 1 169634884 | Intergenic/Unknown,Transversion Substitution |
| RS12731991 |  | 0.30 (T) | chr. 1 169699120 | Intergenic/Unknown,Transversion Substitution |
| RS1687064 | *BAT2D1* | 0.19 (C) | chr. 1 169777663 | Silent Mutation,Transition Substitution |
| RS10753138 | *BAT2D1* | 0.48 (C) | chr. 1 169798145 | Intron,Transversion Substitution |
| RS12035960 | *MYOC* | 0.10 (T) | chr. 1 169881829 | Intron,Transition Substitution |
| RS10913530 | *VAMP4* | 0.26 (A) | chr. 1 169946581 | Intron,Transition Substitution |
| RS7556644 |  | 0.32 (C) | chr. 1 169979491 | Intergenic/Unknown,Transversion Substitution |
| RS2232816 | *KIAA0859* | 0.23 (G) | chr. 1 170019662 | Mis-sense Mutation,Transition Substitution |
| RS764179 |  | 0.39 (A) | chr. 1 170067031 | Intergenic/Unknown,Transition Substitution |
| RS6673646 | *DNM3* | 0.48 (T) | chr. 1 170163821 | Intron,Transition Substitution |
| RS2104037 | *DNM3* | 0.43 (C) | chr. 1 170253110 | Intron, Transition Substitution |
| RS17346473 | *DNM3* | 0.22 (G) | chr. 1 170349716 | Intron,Transition Substitution |
| RS2422075 | *DNM3* | 0.16 (C) | chr. 1 170500091 | Intron,Transition Substitution |
| RS6424865 | *DNM3* | 0.42 (G) | chr. 1 170557555 | Intron,Transition Substitution |
| RS7546252 | *DNM3* | 0.29 (A) | chr. 1 170634933 | Intron,Transition Substitution |
| RS3177676 | *C1orf105* | 0.24 (G) | chr. 1 170704215 | Mis-sense Mutation,Transition Substitution |
| RS2051628 |  | 0.18 (A) | chr. 1 170717013 | Intergenic/Unknown,Transversion Substitution |
| RS2239816 | *C1orf9* | 0.31 (C) | chr. 1 170768266 | Mis-sense Mutation,Transition Substitution |
| RS2285664 | *C1orf9* | 0.28 (G) | chr. 1 170814088 | Silent Mutation,Transition Substitution |
| RS929087 | *FASLG* | 0.42 (A) | chr. 1 170898680 | Intron,Transition Substitution |
| RS12125542 |  | 0.86 (C) | chr. 1 170993417 | Intergenic/Unknown,Transversion Substitution |
| RS12066143 |  | 0.28 (T) | chr. 1 171081478 | Intergenic/Unknown,Transition Substitution |
| RS7517726 |  | 0.37 (G) | chr. 1 171180200 | Intergenic/Unknown,Transversion Substitution |
| RS2223519 |  | 0.85 (T) | chr. 1 171241087 | Intergenic/Unknown,Transversion Substitution |
| RS723858 | *TNFSF18* | 0.23 (A) | chr. 1 171284886 | Intron,Transversion Substitution |
| RS3961997 |  | 0.35 (G) | chr. 1 171372661 | Intergenic/Unknown,Transversion Substitution |
| RS1234314 |  | 0.43 (G) | chr. 1 171444015 | Intergenic/Unknown,Transversion Substitution |
| RS10912580 |  | 0.21 (G) | chr. 1 171523173 | Intergenic/Unknown,Transition Substitution |
| RS10912594 |  | 0.38 (G) | chr. 1 171585287 | Intergenic/Unknown,Transversion Substitution |
| RS10912594 |  | 0.38 (G) | chr. 1 171585287 | Intergenic/Unknown,Transversion Substitution |
| RS1418191 |  | 0.15 (C) | chr. 1 171629080 | Intergenic/Unknown,Transition Substitution |
| RS7540065 | *PRDX6* | 0.24 (A) | chr. 1 171715659 | Intron,Transition Substitution |
| RS16846206 | *SLC9A11* | 0.25 (C) | chr. 1 171783494 | Mis-sense Mutation,Transversion Substitution |
| RS12561820 |  | 0.28 (T) | chr. 1 171840275 | Intergenic/Unknown,Transition Substitution |
| RS10912660 | *ANKRD45* | 0.28 (T) | chr. 1 171904141 | Intron,Transversion Substitution |
| RS2273366 | *KLHL20* | 0.34 (G) | chr. 1 171991565 | Intron,Transition Substitution |
| RS7515721 | *CENPL* | 0.34 (G) | chr. 1 172040279 | Intron,Transversion Substitution |
| RS9425756 | *DARS2* | 0.29 (T) | chr. 1 172083209 | Intron,Transition Substitution |
| RS9286895 | *GAS5,ZBTB37,SNORD47,SNORD44* | 0.36 (G) | chr. 1 172109090 | Intron,Transition Substitution |
| RS767053 | *RC3H1* | 0.13 (A) | chr. 1 172170360 | UTR 5,Transition Substitution |
| RS10798301 | *RC3H1* | 0.25 (G) | chr. 1 172215955 | Intron,Transition Substitution |
| RS10494491 |  | 0.13 (C) | chr. 1 172269774 | Intergenic/Unknown,Transversion Substitution |
| RS12085290 |  | 0.43 (G) | chr. 1 172360460 | Intergenic/Unknown,Transversion Substitution |
| RS1890881 | *RABGAP1L* | 0.12 (C) | chr. 1 172443546 | Intron,Transition Substitution |
| RS6681390 | *RABGAP1L* | 0.57 (A) | chr. 1 172497092 | Intron,Transition Substitution |
| RS12097252 | *RABGAP1L* | 0.25 (T) | chr. 1 172554135 | Intron,Transition Substitution |
| RS10912773 | *RABGAP1L* | 0.39 (A) | chr. 1 172610328 | Intron,Transition Substitution |
| RS16847012 | *RABGAP1L* | 0.87 (T) | chr. 1 172666276 | Intron,Transition Substitution |
| RS6674239 | *RABGAP1L* | 0.88 (C) | chr. 1 172699902 | Intron,Transition Substitution |
| RS909533 | *RABGAP1L* | 0.25 (C) | chr. 1 172767189 | Intron,Transversion Substitution |
| RS2072758 | *RABGAP1L* | 0.12 (T) | chr. 1 172792540 | Intron,Transition Substitution |
| RS12079879 | *RABGAP1L* | 0.24 (A) | chr. 1 172807638 | Intron,Transversion Substitution |
| RS2760058 | *RABGAP1L* | 0.24 (T) | chr. 1 172877081 | Intron,Transition Substitution |
| RS459078 | *RABGAP1L* | 0.28 (T) | chr. 1 172969533 | Intron,Transition Substitution |
| RS6658917 | *RABGAP1L* | 0.90 (A) | chr. 1 173057434 | Intron,Transition Substitution |
| RS1231829 | *RABGAP1L* | 0.28 (G) | chr. 1 173089655 | Intron,Transition Substitution |
| RS6688577 | *RABGAP1L* | 0.40 (T) | chr. 1 173150784 | Intron,Transition Substitution |
| RS1034462 | *MRPS14,CACYBP* | 0.24 (C) | chr. 1 173246946 | UTR 3,Transition Substitution |
| RS10912867 | *CACYBP,MRPS14* | 0.25 (A) | chr. 1 173251449 | Intron,Transition Substitution |
| RS10489329 |  | 0.27 (T) | chr. 1 173289029 | Intergenic/Unknown,Transversion Substitution |
| RS2072032 | *TNN* | 0.48 (A) | chr. 1 173313412 | Mis-sense Mutation,Transition Substitution |
| RS16847812 | *TNN* | 0.86 (G) | chr. 1 173316002 | Mis-sense Mutation,Transition Substitution |
| RS6696455 | *TNN* | 0.53 (T) | chr. 1 173354352 | Mis-sense Mutation,Transition Substitution |
| RS2269650 | *KIAA0040* | 0.28 (T) | chr. 1 173396314 | Mis-sense Mutation,Transversion Substitution |
| RS7517699 |  | 0.37 (A) | chr. 1 173472276 | Intergenic/Unknown,Transition Substitution |
| RS6688599 | *TNR* | 0.21 (T) | chr. 1 173565175 | Intron,Transition Substitution |
| RS9286908 | *TNR* | 0.85 (A) | chr. 1 173576066 | Intron,Transition Substitution |
| RS10912977 |  | 0.26 (T) | chr. 1 173665736 | Intergenic/Unknown,Transition Substitution |
| RS859379 |  | 0.41 (C) | chr. 1 173745306 | Intergenic/Unknown,Transition Substitution |
| RS6692436 |  | 0.14 (C) | chr. 1 173837353 | Intergenic/Unknown,Transition Substitution |
| RS12030656 |  | 0.44 (T) | chr. 1 173927353 | Intergenic/Unknown,Transition Substitution |
| RS7528236 |  | 0.31 (G) | chr. 1 174019909 | Intergenic/Unknown,Transition Substitution |
| RS578140 |  | 0.28 (G) | chr. 1 174114711 | Intergenic/Unknown,Transition Substitution |
| RS1357337 | *RFWD2* | 0.12 (C) | chr. 1 174197560 | Intron,Transition Substitution |
| RS17351808 | *RFWD2* | 0.10 (C) | chr. 1 174279332 | Intron,Transition Substitution |
| RS2481641 | *RFWD2* | 0.21 (A) | chr. 1 174325405 | Intron,Transversion Substitution |
| RS10913144 | *RFWD2* | 0.48 (G) | chr. 1 174382989 | Intron,Transversion Substitution |
| RS580978 | *RFWD2* | 0.12 (G) | chr. 1 174441756 | Intron,Transition Substitution |
| RS1503122 |  | 0.11 (G) | chr. 1 174516332 | Intergenic/Unknown,Transversion Substitution |
| RS10913158 |  | 0.18 (A) | chr. 1 174578220 | Intergenic/Unknown,Transition Substitution |
| RS2504485 |  | 0.24 (A) | chr. 1 174660932 | Intergenic/Unknown,Transition Substitution |
| RS6425386 | *PAPPA2* | 0.34 (T) | chr. 1 174712325 | Intron,Transversion Substitution |
| RS2206506 | *PAPPA2* | 0.28 (T) | chr. 1 174769245 | Intron,Transversion Substitution |
| RS12073069 | *PAPPA2* | 0.60 (T) | chr. 1 174860479 | Intron,Transition Substitution |
| RS10753136 | *PAPPA2* | 0.40 (A) | chr. 1 174938244 | Intron,Transition Substitution |
| RS7529487 | *PAPPA2* | 0.42 (T) | chr. 1 174993725 | Intron,Transition Substitution |
| RS10913257 | *PAPPA2* | 0.41 (T) | chr. 1 175045233 | Intron,Transversion Substitution |
| RS17312648 |  | 0.81 (T) | chr. 1 175055397 | Intron,Transition Substitution |
| RS2281180 | *ASTN1* | 0.20 (G) | chr. 1 175130459 | Mis-sense Mutation,Transversion Substitution |
| RS17313493 | *ASTN1* | 0.13 (C) | chr. 1 175172927 | Intron,Transition Substitution |
| RS7536949 | *ASTN1* | 0.41 (A) | chr. 1 175252821 | Intron,Transition Substitution |
| RS17379768 | *ASTN1* | 0.05 (T) | chr. 1 175340896 | Intron,Transversion Substitution |
| RS17380303 | *FAM5B* | 0.10 (T) | chr. 1 175420103 | Intron,Transition Substitution |
| RS3176443 | *FAM5B* | 0.20 (G) | chr. 1 175514477 | Mis-sense Mutation,Transversion Substitution |
| RS17660139 |  | 0.13 (A) | chr. 1 175609904 | Intergenic/Unknown,Transition Substitution |
| RS715359 |  | 0.26 (C) | chr. 1 175697791 | Intergenic/Unknown,Transition Substitution |
| RS2038489 |  | 0.34 (C) | chr. 1 175797480 | Intergenic/Unknown,Transition Substitution |
| RS1890035 |  | 0.44 (C) | chr. 1 175833282 | Intergenic/Unknown,Transversion Substitution |
| RS1923624 |  | 0.81 (G) | chr. 1 175902502 | Intergenic/Unknown,Transition Substitution |
| RS2902197 |  | 0.13 (T) | chr. 1 175981091 | Intergenic/Unknown,Transition Substitution |
| RS1854288 |  | 0.38 (A) | chr. 1 176069710 | Intergenic/Unknown,Transition Substitution |
| RS3813648 | *SEC16B* | 0.81 (G) | chr. 1 176169105 | Intron,Transition Substitution |
| RS943762 | *SEC16B* | 0.09 (T) | chr. 1 176175475 | Mis-sense Mutation,Transition Substitution |
| RS12409266 |  | 0.17 (T) | chr. 1 176237621 | Intergenic/Unknown,Transition Substitution |
| RS12405310 |  | 0.13 (A) | chr. 1 176293776 | Intergenic/Unknown,Transition Substitution |
| RS10494510 | *RASAL2* | 0.20 (G) | chr. 1 176341204 | Intron,Transition Substitution |
| RS389563 | *RASAL2* | 0.27 (G) | chr. 1 176429767 | Intron,Transversion Substitution |
| RS7547504 | *RASAL2* | 0.22 (C) | chr. 1 176467935 | Intron,Transversion Substitution |
| RS1994233 | *RASAL2* | 0.13 (G) | chr. 1 176536446 | Intron,Transition Substitution |
| RS1048418 | *TOR1AIP2* | 0.89 (C) | chr. 1 176562905 | Intron,Transition Substitution |
| RS12022646 | *RASAL2* | 0.13 (G) | chr. 1 176622444 | Intron,Transition Substitution |
| RS2862379 | *RASAL2* | 0.13 (A) | chr. 1 176709529 | UTR 3,Transversion Substitution |
| RS2746325 | *FLJ44005* | 0.28 (T) | chr. 1 176730214 | Mis-sense Mutation,Transition Substitution |
| RS12141152 |  | 0.67 (C) | chr. 1 176780788 | Intron,Transition Substitution |
| RS10913574 |  | 0.36 (C) | chr. 1 176803105 | Intergenic/Unknown,Transversion Substitution |
| RS12028478 |  | 0.75 (A) | chr. 1 176899717 | Intergenic/Unknown,Transition Substitution |
| RS10798630 |  | 0.38 (T) | chr. 1 176958468 | Intergenic/Unknown,Transition Substitution |
| RS16853350 | *RALGPS2* | 0.19 (G) | chr. 1 177031771 | Intron,Transversion Substitution |
| RS3766642 | *ANGPTL1,RALGPS2* | 0.12 (A) | chr. 1 177080154 | Intron,Transition Substitution |
| RS3753535 | *ANGPTL1,RALGPS2* | 0.28 (C) | chr. 1 177103594 | Intron,Transition Substitution |
| RS2031470 |  | 0.28 (T) | chr. 1 177209635 | Intergenic/Unknown,Transition Substitution |
| RS10913673 | *FAM20B* | 0.38 (G) | chr. 1 177263036 | Intron,Transition Substitution |
| RS3845403 |  | 0.25 (A) | chr. 1 177296159 | Intergenic/Unknown,Transition Substitution |
| RS3766629 | *FAM20B* | 0.17 (G) | chr. 1 177302052 | Intron,Transversion Substitution |
| RS9724754 | *ABL2* | 0.13 (A) | chr. 1 177351031 | Intron,Transversion Substitution |
| RS12120194 | *ABL2* | 0.10 (G) | chr. 1 177421080 | Intron,Transition Substitution |
| RS2636288 |  | 0.25 (C) | chr. 1 177494468 | Intergenic/Unknown,Transition Substitution |
| RS10913718 | *SOAT1* | 0.48 (A) | chr. 1 177554031 | Intron,Transition Substitution |
| RS10753191 | *SOAT1* | 0.12 (T) | chr. 1 177579375 | Silent Mutation,Transition Substitution |
| RS3753526 | *SOAT1* | 0.06 (G) | chr. 1 177586164 | Silent Mutation,Transversion Substitution |
| RS10753193 |  | 0.11 (A) | chr. 1 177601607 | Intergenic/Unknown,Transition Substitution |
| RS10913766 | *C1orf125* | 0.29 (G) | chr. 1 177683579 | Intron,Transition Substitution |
| RS10494520 | *C1orf125* | 0.09 (A) | chr. 1 177718898 | Silent Mutation,Transition Substitution |
| RS3738423 | *NPHS2* | 0.10 (A) | chr. 1 177800538 | Silent Mutation,Transition Substitution |
| RS6704505 | *TDRD5* | 0.11 (G) | chr. 1 177866624 | Mis-sense Mutation,Transition Substitution |
| RS4628468 | *TDRD5* | 0.22 (C) | chr. 1 177871487 | Silent Mutation,Transition Substitution |
| RS11578278 | *TDRD5* | 0.37 (T) | chr. 1 177926152 | Intron,Transition Substitution |
| RS3843266 |  | 0.25 (A) | chr. 1 177957508 | Intergenic/Unknown,Transition Substitution |
| RS17370139 | *C1orf76* | 0.23 (G) | chr. 1 177998076 | Intron,Transition Substitution |
| RS2236589 | *C1orf76* | 0.43 (A) | chr. 1 178050545 | UTR 3,Transversion Substitution |
| RS1281411 | *TOR1AIP2* | 0.48 (C) | chr. 1 178081080 | UTR 3,Transversion Substitution |
| RS1281378 | *TOR1AIP2,TOR1AIP1* | 0.43 (T) | chr. 1 178118697 | Mis-sense Mutation,Transition Substitution |
| RS609521 | *TOR1AIP1* | 0.44 (C) | chr. 1 178143611 | Mis-sense Mutation,Transversion Substitution |
| RS627897 | *TOR1AIP1* | 0.10 (G) | chr. 1 178153748 | Silent Mutation,Transition Substitution |
| RS507603 |  | 0.24 (A) | chr. 1 178163693 | Intergenic/Unknown,Transversion Substitution |
| RS2501617 | *CEP350* | 0.39 (T) | chr. 1 178226992 | Intron,Transition Substitution |
| RS9425859 | *CEP350* | 0.27 (A) | chr. 1 178315732 | Intron,Transition Substitution |
| RS3767202 | *CEP350* | 0.39 (T) | chr. 1 178346738 | Intron,Transversion Substitution |
| RS3767199 | *QSOX1* | 0.32 (T) | chr. 1 178391290 | Intron,Transversion Substitution |
| RS3767170 | *QSOX1* | 0.29 (C) | chr. 1 178424190 | Intron,Transition Substitution |
| RS6425616 | *LHX4* | 0.47 (G) | chr. 1 178467807 | Intron,Transition Substitution |
| RS17372142 | *LHX4* | 0.08 (C) | chr. 1 178505065 | Intron,Transversion Substitution |
| RS2764468 | *ACBD6* | 0.07 (G) | chr. 1 178536393 | Intron,Transversion Substitution |
| RS17372362 | *ACBD6* | 0.21 (C) | chr. 1 178590312 | Intron,Transition Substitution |
| RS4076403 | *ACBD6* | 0.41 (A) | chr. 1 178661103 | Intron,Transition Substitution |
| RS3806300 |  | 0.12 (C) | chr. 1 178738993 | Intergenic/Unknown,Transition Substitution |
| RS12402575 | *XPR1* | 0.21 (C) | chr. 1 178872209 | Intron,Transition Substitution |
| RS10914077 | *XPR1* | 0.41 (G) | chr. 1 178952544 | Intron,Transition Substitution |
| RS3013633 | *XPR1* | 0.76 (G) | chr. 1 179030742 | Intron,Transition Substitution |
| RS1061015 | *XPR1* | 0.43 (T) | chr. 1 179120342 | UTR 3,Transition Substitution |
| RS6672415 |  | 0.38 (T) | chr. 1 179147771 | Intergenic/Unknown,Transition Substitution |
| RS3747957 | *STX6* | 0.43 (A) | chr. 1 179220476 | Silent Mutation,Transition Substitution |
| RS17374409 | *STX6* | 0.09 (C) | chr. 1 179239541 | Intron,Transversion Substitution |
| RS2282348 |  | 0.11 (T) | chr. 1 179294548 | Intergenic/Unknown,Transversion Substitution |
| RS6671643 |  | 0.43 (G) | chr. 1 179384857 | Intergenic/Unknown,Transition Substitution |
| RS10732972 |  | 0.19 (C) | chr. 1 179472345 | Intergenic/Unknown,Transition Substitution |
| RS7525299 |  | 0.46 (A) | chr. 1 179562735 | Intergenic/Unknown,Transition Substitution |
| RS12039057 |  | 0.75 (A) | chr. 1 179604962 | Intergenic/Unknown,Transition Substitution |
| RS12066616 |  | 0.31 (T) | chr. 1 179695384 | Intergenic/Unknown,Transition Substitution |
| RS1320515 | *CACNA1E* | 0.18 (A) | chr. 1 179786677 | Intron,Transition Substitution |
| RS199939 | *CACNA1E* | 0.20 (T) | chr. 1 179862602 | Intron,Transition Substitution |
| RS16858006 | *CACNA1E* | 0.09 (A) | chr. 1 179936797 | Intron,Transversion Substitution |
| RS199930 | *CACNA1E* | 0.79 (C) | chr. 1 180007924 | Silent Mutation,Transition Substitution |
| RS10489642 |  | 0.12 (G) | chr. 1 180099184 | Intergenic/Unknown,Transition Substitution |
| RS498212 |  | 0.31 (C) | chr. 1 180136840 | Intergenic/Unknown,Transition Substitution |
| RS16858394 |  | 0.19 (G) | chr. 1 180204310 | Intergenic/Unknown,Transversion Substitution |
| RS1281311 |  | 0.18 (G) | chr. 1 180276664 | Intergenic/Unknown,Transition Substitution |
| RS12409614 |  | 0.40 (A) | chr. 1 180352064 | Intergenic/Unknown,Transition Substitution |
| RS16858639 |  | 0.87 (G) | chr. 1 180448547 | Intergenic/Unknown,Transition Substitution |
| RS2985425 |  | 0.27 (G) | chr. 1 180543771 | Intergenic/Unknown,Transition Substitution |
| RS1058111 | *GLUL* | 0.48 (G) | chr. 1 180623022 | Silent Mutation,Transition Substitution |
| RS267899 | *RGSL2* | 0.32 (A) | chr. 1 180712979 | Intron,Transition Substitution |
| RS1048260 | *RNASEL* | 0.23 (G) | chr. 1 180809474 | UTR 3,Transversion Substitution |
| RS597880 | *RGS16* | 0.35 (C) | chr. 1 180839498 | Intron,Transition Substitution |
| RS2023596 | *RGS8* | 0.21 (G) | chr. 1 180889634 | Intron,Transition Substitution |
| RS2482806 |  | 0.32 (C) | chr. 1 180950710 | Intergenic/Unknown,Transversion Substitution |
| RS7554927 | *NPL* | 0.11 (T) | chr. 1 181033121 | Intron,Transversion Substitution |
| RS3130492 | *DHX9* | 0.49 (A) | chr. 1 181120640 | Intron,Transition Substitution |
| RS12025917 | *C1orf14* | 0.06 (C) | chr. 1 181182426 | Intron,Transition Substitution |
| RS10911168 |  | 0.90 (C) | chr. 1 181195911 | Intergenic/Unknown,Transition Substitution |
| RS4652769 | *LAMC1* | 0.47 (C) | chr. 1 181275819 | Intron,Transition Substitution |
| RS1360704 | *LAMC1* | 0.38 (C) | chr. 1 181366003 | Intron,Transition Substitution |
| RS2147578 | *LAMC1* | 0.65 (G) | chr. 1 181374322 | Intron,Transversion Substitution |
| RS2296303 | *LAMC2* | 0.08 (G) | chr. 1 181468593 | Mis-sense Mutation,Transversion Substitution |
| RS607332 | *NMNAT2* | 0.42 (A) | chr. 1 181519836 | Intron,Transition Substitution |
| RS2811557 | *NMNAT2* | 0.35 (T) | chr. 1 181596878 | Intron,Transition Substitution |
| RS2702199 |  | 0.37 (T) | chr. 1 181668503 | Intergenic/Unknown,Transition Substitution |
| RS789169 | *SMG7* | 0.07 (G) | chr. 1 181753482 | Silent Mutation,Transition Substitution |
| RS3843293 | *NCF2* | 0.44 (T) | chr. 1 181806072 | Intron,Transversion Substitution |
| RS1174657 | *APOBEC4,RGL1* | 0.46 (C) | chr. 1 181883549 | Intron,Transition Substitution |
| RS1174658 | *APOBEC4,RGL1* | 0.46 (G) | chr. 1 181883728 | Intron,Transition Substitution |
| RS16861394 | *RGL1* | 0.92 (G) | chr. 1 181884316 | Mis-sense Mutation,Transition Substitution |
| RS12092963 | *RGL1* | 0.14 (T) | chr. 1 181938444 | Intron,Transition Substitution |
| RS1926834 | *RGL1* | 0.40 (T) | chr. 1 182005986 | Intron,Transition Substitution |
| RS12137911 | *RGL1* | 0.60 (T) | chr. 1 182080126 | Intron,Transition Substitution |
| RS12098028 | *RGL1* | 0.73 (C) | chr. 1 182143112 | Intron,Transversion Substitution |
| RS7515190 | *GLT25D2* | 0.41 (G) | chr. 1 182206937 | Intron,Transversion Substitution |
| RS10752928 | *GLT25D2* | 0.39 (G) | chr. 1 182263614 | Intron,Transition Substitution |
| RS1046934 | *C1orf19* | 0.38 (C) | chr. 1 182290152 | Mis-sense Mutation,Transversion Substitution |
| RS3845468 |  | 0.47 (G) | chr. 1 182352656 | Intergenic/Unknown,Transition Substitution |
| RS12134464 |  | 0.39 (C) | chr. 1 182399892 | Intergenic/Unknown,Transversion Substitution |
| RS2161989 |  | 0.15 (T) | chr. 1 182454323 | Intergenic/Unknown,Transversion Substitution |
| RS932993 |  | 0.46 (C) | chr. 1 182550297 | Intergenic/Unknown,Transition Substitution |
| RS1884202 | *C1orf21* | 0.09 (A) | chr. 1 182642734 | Intron,Transition Substitution |
| RS12401841 | *C1orf21* | 0.19 (A) | chr. 1 182739520 | Intron,Transversion Substitution |
| RS6693408 | *C1orf21* | 0.16 (C) | chr. 1 182787351 | Intron,Transversion Substitution |
| RS4651224 | *C1orf21* | 0.36 (T) | chr. 1 182851805 | Intron,Transition Substitution |
| RS4651229 |  | 0.38 (A) | chr. 1 182890182 | Intergenic/Unknown,Transition Substitution |
| RS9425343 | *EDEM3* | 0.37 (C) | chr. 1 182930160 | Mis-sense Mutation,Transversion Substitution |
| RS3736757 | *EDEM3* | 0.45 (A) | chr. 1 182944087 | Silent Mutation,Transition Substitution |
| RS861602 |  | 0.46 (A) | chr. 1 183012191 | Intergenic/Unknown,Transition Substitution |
| RS487675 | *FAM129A* | 0.35 (C) | chr. 1 183067688 | Silent Mutation,Transition Substitution |
| RS532092 | *FAM129A* | 0.42 (A) | chr. 1 183118278 | Intron,Transition Substitution |
| RS234123 | *FAM129A* | 0.27 (C) | chr. 1 183194788 | Intron,Transition Substitution |
| RS10911661 |  | 0.18 (G) | chr. 1 183229372 | Intergenic/Unknown,Transition Substitution |
| RS2378958 | *RNF2* | 0.13 (G) | chr. 1 183309023 | Intron,Transversion Substitution |
| RS1407554 | *C1orf25* | 0.06 (C) | chr. 1 183372890 | Intron,Transversion Substitution |
| RS10489579 | *C1orf26* | 0.40 (G) | chr. 1 183410344 | Mis-sense Mutation,Transition Substitution |
| RS6698109 | *C1orf26* | 0.40 (G) | chr. 1 183438492 | Mis-sense Mutation,Transition Substitution |
| RS12041704 | *C1orf26* | 0.49 (G) | chr. 1 183507097 | Mis-sense Mutation,Transition Substitution |
| RS10174 | *IVNS1ABP,C1orf26* | 0.48 (G) | chr. 1 183533737 | UTR 3,Transition Substitution |
| RS726567 | *IVNS1ABP* | 0.47 (G) | chr. 1 183562377 | Intron,Transition Substitution |
| RS12566660 |  | 0.77 (G) | chr. 1 183657193 | Intergenic/Unknown,Transversion Substitution |
| RS10911744 |  | 0.46 (T) | chr. 1 183745863 | Intergenic/Unknown,Transition Substitution |
| RS971224 |  | 0.43 (A) | chr. 1 183838556 | Intergenic/Unknown,Transition Substitution |
| RS1358871 |  | 0.29 (G) | chr. 1 183930850 | Intergenic/Unknown,Transversion Substitution |
| RS1321670 | *HMCN1* | 0.28 (A) | chr. 1 184006852 | Intron,Transition Substitution |
| RS7556537 | *HMCN1* | 0.49 (C) | chr. 1 184100188 | Intron,Transversion Substitution |
| RS16824765 | *HMCN1* | 0.37 (T) | chr. 1 184172829 | Intron,Transition Substitution |
| RS6665753 | *HMCN1* | 0.43 (C) | chr. 1 184251140 | Silent Mutation,Transition Substitution |
| RS10798035 | *HMCN1* | 0.48 (A) | chr. 1 184317040 | Mis-sense Mutation,Transition Substitution |
| RS17531405 | *HMCN1* | 0.17 (C) | chr. 1 184380475 | Intron,Transversion Substitution |
| RS633606 |  | 0.20 (T) | chr. 1 184470431 | Intergenic/Unknown,Transition Substitution |
| RS3766709 | *TPR* | 0.35 (T) | chr. 1 184562553 | Intron,Transition Substitution |
| RS11806304 | *OCLM,C1orf27* | 0.07 (G) | chr. 1 184639488 | Intron,Transition Substitution |
| RS12402521 | *PDC* | 0.23 (A) | chr. 1 184689144 | Intron,Transition Substitution |
| RS11582183 |  | 0.23 (C) | chr. 1 184757940 | Intergenic/Unknown,Transition Substitution |
| RS11809657 |  | 0.07 (T) | chr. 1 184840353 | Intergenic/Unknown,Transition Substitution |
| RS10911898 |  | 0.23 (T) | chr. 1 184879216 | Intergenic/Unknown,Transversion Substitution |
| RS17588736 |  | 0.16 (A) | chr. 1 184958747 | Intergenic/Unknown,Transition Substitution |
| RS4651329 |  | 0.08 (G) | chr. 1 185043507 | Intergenic/Unknown,Transition Substitution |
| RS2223309 | *PLA2G4A* | 0.09 (A) | chr. 1 185114716 | Intron,Transition Substitution |
| RS726706 | *PLA2G4A* | 0.44 (A) | chr. 1 185139077 | Intron,Transition Substitution |
| RS7555140 | *PLA2G4A* | 0.32 (C) | chr. 1 185184143 | Intron,Transversion Substitution |
| RS17601344 | *PLA2G4A* | 0.06 (T) | chr. 1 185219344 | Intron,Transition Substitution |
| RS10911992 |  | 0.18 (C) | chr. 1 185306087 | Intergenic/Unknown,Transition Substitution |
| RS10912025 |  | 0.15 (T) | chr. 1 185404762 | Intergenic/Unknown,Transversion Substitution |
| RS1116799 |  | 0.42 (C) | chr. 1 185493229 | Intergenic/Unknown,Transition Substitution |
| RS7523068 |  | 0.14 (A) | chr. 1 185577578 | Intergenic/Unknown,Transition Substitution |
| RS6667870 |  | 0.32 (T) | chr. 1 185658532 | Intergenic/Unknown,Transition Substitution |
| RS1538474 |  | 0.22 (C) | chr. 1 185756729 | Intergenic/Unknown,Transition Substitution |
| RS1416478 |  | 0.43 (G) | chr. 1 185840651 | Intergenic/Unknown,Transition Substitution |
| RS3120965 |  | 0.37 (G) | chr. 1 185937965 | Intergenic/Unknown,Transition Substitution |
| RS2495420 |  | 0.06 (G) | chr. 1 186034695 | Intergenic/Unknown,Transition Substitution |
| RS16827680 |  | 0.89 (A) | chr. 1 186119332 | Intergenic/Unknown,Transition Substitution |
| RS1432432 |  | 0.21 (A) | chr. 1 186213763 | Intergenic/Unknown,Transition Substitution |
| RS1923809 |  | 0.22 (G) | chr. 1 186304189 | Intergenic/Unknown,Transversion Substitution |
| RS354264 |  | 0.18 (T) | chr. 1 186398390 | Intergenic/Unknown,Transversion Substitution |
| RS1955245 |  | 0.34 (G) | chr. 1 186491290 | Intergenic/Unknown,Transition Substitution |
| RS4468122 |  | 0.61 (A) | chr. 1 186585283 | Intergenic/Unknown,Transition Substitution |
| RS1489239 |  | 0.30 (A) | chr. 1 186684426 | Intergenic/Unknown,Transition Substitution |
| RS2872064 |  | 0.06 (A) | chr. 1 186771907 | Intergenic/Unknown,Transition Substitution |
| RS1609772 |  | 0.25 (A) | chr. 1 186820222 | Intergenic/Unknown,Transversion Substitution |
| RS1902875 |  | 0.92 (C) | chr. 1 186875734 | Intergenic/Unknown,Transition Substitution |
| RS12758150 |  | 0.18 (T) | chr. 1 186966692 | Intergenic/Unknown,Transition Substitution |
| RS7537197 |  | 0.05 (G) | chr. 1 187039734 | Intergenic/Unknown,Transversion Substitution |
| RS1445665 |  | 0.05 (G) | chr. 1 187101445 | Intergenic/Unknown,Transition Substitution |
| RS10801032 |  | 0.44 (A) | chr. 1 187167768 | Intergenic/Unknown,Transition Substitution |
| RS6675714 |  | 0.36 (A) | chr. 1 187239604 | Intergenic/Unknown,Transition Substitution |
| RS10737614 |  | 0.46 (A) | chr. 1 187319910 | Intergenic/Unknown,Transversion Substitution |
| RS7537785 |  | 0.43 (C) | chr. 1 187383793 | Intergenic/Unknown,Transition Substitution |
| RS10801272 |  | 0.31 (T) | chr. 1 187442374 | Intergenic/Unknown,Transition Substitution |
| RS12130800 |  | 0.44 (T) | chr. 1 187532871 | Intergenic/Unknown,Transition Substitution |
| RS6667196 |  | 0.38 (T) | chr. 1 187602301 | Intergenic/Unknown,Transversion Substitution |
| RS1704589 |  | 0.38 (C) | chr. 1 187674150 | Intergenic/Unknown,Transition Substitution |
| RS269737 |  | 0.43 (G) | chr. 1 187738393 | Intergenic/Unknown,Transversion Substitution |
| RS1848826 |  | 0.35 (A) | chr. 1 187822352 | Intergenic/Unknown,Transversion Substitution |
| RS480025 |  | 0.49 (C) | chr. 1 187897346 | Intergenic/Unknown,Transition Substitution |
| RS7543449 |  | 0.46 (G) | chr. 1 187984282 | Intergenic/Unknown,Transition Substitution |
| RS10919795 |  | 0.16 (T) | chr. 1 188045805 | Intergenic/Unknown,Transversion Substitution |
| RS1568134 |  | 0.83 (G) | chr. 1 188088932 | Intergenic/Unknown,Transition Substitution |
| RS815761 |  | 0.26 (C) | chr. 1 188171798 | Intergenic/Unknown,Transition Substitution |
| RS12145507 |  | 0.83 (A) | chr. 1 188237152 | Intergenic/Unknown,Transition Substitution |
| RS10920424 |  | 0.32 (T) | chr. 1 188302763 | Intergenic/Unknown,Transition Substitution |
| RS7412309 | *FAM5C* | 0.06 (C) | chr. 1 188344664 | Intron,Transition Substitution |
| RS1891586 | *FAM5C* | 0.24 (G) | chr. 1 188430617 | Intron,Transversion Substitution |
| RS1855241 | *FAM5C* | 0.39 (T) | chr. 1 188498748 | Intron,Transition Substitution |
| RS510498 | *FAM5C* | 0.16 (C) | chr. 1 188534730 | Intron,Transition Substitution |
| RS1031076 | *FAM5C* | 0.49 (C) | chr. 1 188629558 | Intron,Transversion Substitution |
| RS814925 |  | 0.48 (A) | chr. 1 188722485 | Intergenic/Unknown,Transition Substitution |
| RS10753949 |  | 0.36 (G) | chr. 1 188811906 | Intergenic/Unknown,Transition Substitution |
| RS17380253 |  | 0.14 (T) | chr. 1 188877974 | Intergenic/Unknown,Transition Substitution |
| RS10920773 |  | 0.43 (T) | chr. 1 188921943 | Intergenic/Unknown,Transition Substitution |
| RS982661 |  | 0.05 (G) | chr. 1 189011413 | Intergenic/Unknown,Transition Substitution |
| RS1370881 |  | 0.35 (C) | chr. 1 189103456 | Intergenic/Unknown,Transition Substitution |
| RS1431142 |  | 0.39 (G) | chr. 1 189201006 | Intergenic/Unknown,Transition Substitution |
| RS1886414 |  | 0.23 (C) | chr. 1 189282724 | Intergenic/Unknown,Transition Substitution |
| RS1454353 |  | 0.38 (T) | chr. 1 189360439 | Intergenic/Unknown,Transition Substitution |
| RS10494650 |  | 0.38 (T) | chr. 1 189448041 | Intergenic/Unknown,Transition Substitution |
| RS3128544 |  | 0.18 (C) | chr. 1 189546888 | Intergenic/Unknown,Transversion Substitution |
| RS2781058 |  | 0.33 (C) | chr. 1 189583271 | Intergenic/Unknown,Transition Substitution |
| RS580700 |  | 0.35 (G) | chr. 1 189676598 | Intergenic/Unknown,Transition Substitution |
| RS1890231 |  | 0.36 (A) | chr. 1 189768016 | Intergenic/Unknown,Transition Substitution |
| RS1415865 |  | 0.25 (T) | chr. 1 189860361 | Intergenic/Unknown,Transition Substitution |
| RS7515753 |  | 0.85 (G) | chr. 1 189946877 | Intergenic/Unknown,Transition Substitution |
| RS2000054 |  | 0.24 (G) | chr. 1 190045294 | Intergenic/Unknown,Transition Substitution |
| RS9427808 |  | 0.34 (G) | chr. 1 190134936 | Intergenic/Unknown,Transition Substitution |
| RS7413609 |  | 0.46 (C) | chr. 1 190228707 | Intergenic/Unknown,Transition Substitution |
| RS2999589 |  | 0.43 (G) | chr. 1 190308010 | Intergenic/Unknown,Transversion Substitution |
| RS10754003 | *RGS18* | 0.40 (A) | chr. 1 190403768 | Intron,Transversion Substitution |
| RS7540236 |  | 0.28 (G) | chr. 1 190500159 | Intergenic/Unknown,Transition Substitution |
| RS1892145 | *RGS21* | 0.30 (T) | chr. 1 190595078 | Intron,Transversion Substitution |
| RS12563358 |  | 0.25 (A) | chr. 1 190689628 | Intergenic/Unknown,Transition Substitution |
| RS1323298 |  | 0.20 (T) | chr. 1 190786488 | Intergenic/Unknown,Transition Substitution |
| RS12044559 | *RGS13* | 0.43 (C) | chr. 1 190875891 | Intron,Transition Substitution |
| RS12728622 |  | 0.49 (A) | chr. 1 190971320 | Intergenic/Unknown,Transition Substitution |
| RS710146 |  | 0.26 (C) | chr. 1 191062042 | Intergenic/Unknown,Transition Substitution |
| RS2370025 |  | 0.45 (A) | chr. 1 191158646 | Intergenic/Unknown,Transition Substitution |
| RS12132305 |  | 0.06 (A) | chr. 1 191243704 | Intergenic/Unknown,Transition Substitution |
| RS3820417 | *UCHL5* | 0.23 (C) | chr. 1 191267832 | Intron,Transition Substitution |
| RS12131585 | *UCHL5,TROVE2* | 0.08 (G) | chr. 1 191301636 | Intron,Transition Substitution |
| RS912071 | *GLRX2* | 0.28 (C) | chr. 1 191341731 | UTR 5,Transition Substitution |
| RS2370029 | *CDC73* | 0.27 (G) | chr. 1 191389070 | Intron,Transition Substitution |
| RS1325197 | *B3GALT2,CDC73* | 0.26 (T) | chr. 1 191418940 | Intron,Transition Substitution |
| RS11583414 | *CDC73* | 0.12 (G) | chr. 1 191485466 | Intron,Transversion Substitution |
| RS12037055 |  | 0.24 (C) | chr. 1 191583311 | Intergenic/Unknown,Transition Substitution |
| RS16829482 |  | 0.21 (T) | chr. 1 191657921 | Intergenic/Unknown,Transversion Substitution |
| RS2093852 |  | 0.48 (C) | chr. 1 191729492 | Intergenic/Unknown,Transition Substitution |
| RS10494688 |  | 0.38 (A) | chr. 1 191770628 | Intergenic/Unknown,Transition Substitution |
| RS6428182 |  | 0.17 (G) | chr. 1 191859017 | Intergenic/Unknown,Transition Substitution |
| RS4657759 |  | 0.47 (G) | chr. 1 191937555 | Intergenic/Unknown,Transition Substitution |
| RS1327445 |  | 0.42 (A) | chr. 1 192031184 | Intergenic/Unknown,Transversion Substitution |
| RS670747 |  | 0.24 (G) | chr. 1 192125648 | Intergenic/Unknown,Transversion Substitution |
| RS12120715 |  | 0.82 (C) | chr. 1 192180063 | Intergenic/Unknown,Transversion Substitution |
| RS12749556 |  | 0.22 (A) | chr. 1 192231836 | Intergenic/Unknown,Transition Substitution |
| RS1830680 |  | 0.14 (G) | chr. 1 192311587 | Intergenic/Unknown,Transition Substitution |
| RS2506058 |  | 0.13 (T) | chr. 1 192372903 | Intergenic/Unknown,Transition Substitution |
| RS6670222 |  | 0.85 (G) | chr. 1 192414306 | Intergenic/Unknown,Transition Substitution |
| RS653166 |  | 0.11 (T) | chr. 1 192497072 | Intergenic/Unknown,Transversion Substitution |
| RS6683050 |  | 0.22 (T) | chr. 1 192585763 | Intergenic/Unknown,Transversion Substitution |
| RS6700592 |  | 0.32 (G) | chr. 1 192658650 | Intergenic/Unknown,Transversion Substitution |
| RS12118823 |  | 0.09 (A) | chr. 1 192743596 | Intergenic/Unknown,Transition Substitution |
| RS3001177 |  | 0.93 (T) | chr. 1 192788170 | Intergenic/Unknown,Transversion Substitution |
| RS3009350 |  | 0.28 (T) | chr. 1 192869230 | Intergenic/Unknown,Transition Substitution |
| RS1929230 |  | 0.28 (C) | chr. 1 192965181 | Intergenic/Unknown,Transition Substitution |
| RS17645982 |  | 0.24 (A) | chr. 1 193038510 | Intergenic/Unknown,Transition Substitution |
| RS339553 |  | 0.42 (A) | chr. 1 193134533 | Intergenic/Unknown,Transition Substitution |
| RS339577 |  | 0.30 (T) | chr. 1 193197164 | Intergenic/Unknown,Transversion Substitution |
| RS1538549 |  | 0.28 (A) | chr. 1 193271678 | Intergenic/Unknown,Transition Substitution |
| RS1292487 |  | 0.82 (A) | chr. 1 193351159 | Intergenic/Unknown,Transition Substitution |
| RS822456 |  | 0.33 (T) | chr. 1 193361802 | Intergenic/Unknown,Transition Substitution |
| RS10921817 |  | 0.20 (A) | chr. 1 193452046 | Intergenic/Unknown,Transversion Substitution |
| RS7416696 |  | 0.21 (T) | chr. 1 193533946 | Intergenic/Unknown,Transition Substitution |
| RS12409559 |  | 0.48 (C) | chr. 1 193558579 | Intergenic/Unknown,Transversion Substitution |
| RS1324713 |  | 0.27 (C) | chr. 1 193643558 | Intergenic/Unknown,Transition Substitution |
| RS1416748 |  | 0.18 (A) | chr. 1 193739753 | Intergenic/Unknown,Transition Substitution |
| RS2790083 |  | 0.35 (T) | chr. 1 193827463 | Intergenic/Unknown,Transition Substitution |
| RS10801443 |  | 0.15 (A) | chr. 1 193913624 | Intergenic/Unknown,Transversion Substitution |
| RS3010158 |  | 0.38 (G) | chr. 1 194008718 | Intergenic/Unknown,Transition Substitution |
| RS10921969 |  | 0.67 (C) | chr. 1 194103547 | Intergenic/Unknown,Transition Substitution |
| RS2923910 |  | 0.33 (A) | chr. 1 194150069 | Intergenic/Unknown,Transition Substitution |
| RS10922237 |  | 0.56 (G) | chr. 1 194178359 | Intergenic/Unknown,Transversion Substitution |
| RS2923909 |  | 0.32 (T) | chr. 1 194204949 | Intergenic/Unknown,Transversion Substitution |
| RS3937673 |  | 0.43 (C) | chr. 1 194288167 | Intergenic/Unknown,Transition Substitution |
| RS10754178 |  | 0.07 (C) | chr. 1 194386917 | Intergenic/Unknown,Transition Substitution |
| RS7546387 |  | 0.15 (T) | chr. 1 194446269 | Intergenic/Unknown,Transition Substitution |
| RS2477354 | *KCNT2* | 0.16 (A) | chr. 1 194505991 | Intron,Transition Substitution |
| RS12407399 | *KCNT2* | 0.08 (C) | chr. 1 194558600 | Intron,Transition Substitution |
| RS3927686 | *KCNT2* | 0.30 (T) | chr. 1 194652272 | Intron,Transition Substitution |
| RS12065519 | *KCNT2* | 0.30 (G) | chr. 1 194733999 | Intron,Transition Substitution |
| RS4294368 | *KCNT2* | 0.07 (C) | chr. 1 194814311 | Intron,Transition Substitution |
| RS1329427 | *CFH* | 0.65 (C) | chr. 1 194971182 | Intron,Transition Substitution |
| RS438781 | *CFHR1* | 0.46 (T) | chr. 1 195062863 | Intron,Transversion Substitution |
| RS10801575 |  | 0.36 (T) | chr. 1 195119404 | Intergenic/Unknown,Transition Substitution |
| RS3790414 | *CFHR2* | 0.18 (A) | chr. 1 195186922 | Intron,Transversion Substitution |
| RS10922153 | *CFHR5* | 0.44 (T) | chr. 1 195245238 | UTR 3,Transversion Substitution |
| RS5998 | *F13B* | 0.49 (A) | chr. 1 195276421 | Silent Mutation,Transition Substitution |
| RS6003 | *F13B* | 0.08 (C) | chr. 1 195297644 | Mis-sense Mutation,Transition Substitution |
| RS6676084 | *ASPM* | 0.38 (T) | chr. 1 195360653 | Silent Mutation,Transition Substitution |
| RS4350226 | *ZBTB41* | 0.08 (G) | chr. 1 195399001 | Intron,Transition Substitution |
| RS10922187 |  | 0.33 (C) | chr. 1 195454228 | Intergenic/Unknown,Transition Substitution |
| RS489839 | *CRB1* | 0.24 (G) | chr. 1 195505472 | Intron,Transversion Substitution |
| RS570845 | *CRB1* | 0.38 (T) | chr. 1 195559372 | Intron,Transversion Substitution |
| RS2759656 | *CRB1* | 0.25 (A) | chr. 1 195619592 | Intron,Transition Substitution |
| RS10754224 | *DENND1B,C1orf218* | 0.23 (A) | chr. 1 195779412 | Intron,Transversion Substitution |
| RS1362939 | *DENND1B* | 0.23 (C) | chr. 1 195846707 | Intron,Transition Substitution |
| RS6691216 | *DENND1B* | 0.46 (C) | chr. 1 195925886 | Intron,Transition Substitution |
| RS1891497 | *DENND1B* | 0.23 (A) | chr. 1 195926178 | Intron,Transition Substitution |
| RS16841912 | *DENND1B* | 0.20 (C) | chr. 1 195969099 | Intron,Transversion Substitution |
| RS1775468 |  | 0.20 (C) | chr. 1 196020687 | Intergenic/Unknown,Transition Substitution |
| RS1039287 |  | 0.12 (A) | chr. 1 196088494 | Intergenic/Unknown,Transversion Substitution |
| RS1274715 |  | 0.60 (C) | chr. 1 196126239 | Intergenic/Unknown,Transition Substitution |
| RS12046958 | *LHX9* | 0.19 (C) | chr. 1 196163351 | Silent Mutation,Transition Substitution |
| RS10919696 |  | 0.25 (G) | chr. 1 196195162 | Intergenic/Unknown,Transition Substitution |
| RS12046773 |  | 0.40 (A) | chr. 1 196227727 | Intergenic/Unknown,Transversion Substitution |
| RS2093531 |  | 0.14 (A) | chr. 1 196278020 | Intergenic/Unknown,Transition Substitution |
| RS12135790 |  | 0.92 (T) | chr. 1 196345982 | Intergenic/Unknown,Transversion Substitution |
| RS3814322 |  | 0.12 (T) | chr. 1 196405759 | Intergenic/Unknown,Transversion Substitution |
| RS2884687 |  | 0.25 (A) | chr. 1 196461922 | Intergenic/Unknown,Transversion Substitution |
| RS6679100 | *NEK7* | 0.37 (G) | chr. 1 196555332 | UTR 3,Transition Substitution |
| RS16842978 |  | 0.86 (A) | chr. 1 196635746 | Intergenic/Unknown,Transversion Substitution |
| RS12067964 |  | 0.90 (C) | chr. 1 196694799 | Intergenic/Unknown,Transition Substitution |
| RS2257593 | *ATP6V1G3* | 0.23 (T) | chr. 1 196761311 | Intron,Transversion Substitution |
| RS12144380 |  | 0.23 (C) | chr. 1 196831120 | Intergenic/Unknown,Transition Substitution |
| RS1998843 | *PTPRC* | 0.40 (T) | chr. 1 196898979 | Intron,Transition Substitution |
| RS6683595 | *PTPRC* | 0.11 (T) | chr. 1 196969929 | Intron,Transition Substitution |
| RS2759643 |  | 0.27 (T) | chr. 1 197031259 | Intergenic/Unknown,Transversion Substitution |
| RS1407683 |  | 0.40 (A) | chr. 1 197119434 | Intergenic/Unknown,Transition Substitution |
| RS7539177 |  | 0.17 (T) | chr. 1 197213084 | Intergenic/Unknown,Transversion Substitution |
| RS322881 |  | 0.43 (T) | chr. 1 197301939 | Intergenic/Unknown,Transition Substitution |
| RS1384444 |  | 0.42 (T) | chr. 1 197393696 | Intergenic/Unknown,Transition Substitution |
| RS7551857 |  | 0.42 (C) | chr. 1 197462208 | Intergenic/Unknown,Transition Substitution |
| RS6673652 |  | 0.78 (T) | chr. 1 197559958 | Intergenic/Unknown,Transition Substitution |
| RS657714 |  | 0.33 (G) | chr. 1 197622842 | Intergenic/Unknown,Transition Substitution |
| RS2786744 |  | 0.34 (G) | chr. 1 197828525 | Intergenic/Unknown,Transition Substitution |
| RS10919736 |  | 0.34 (A) | chr. 1 197901221 | Intergenic/Unknown,Transition Substitution |
| RS17718224 |  | 0.92 (C) | chr. 1 197997395 | Intergenic/Unknown,Transition Substitution |
| RS12092243 |  | 0.94 (T) | chr. 1 198094713 | Intergenic/Unknown,Transition Substitution |
| RS1991245 |  | 0.38 (A) | chr. 1 198191929 | Intergenic/Unknown,Transition Substitution |
| RS2737621 | *NR5A2* | 0.15 (C) | chr. 1 198264026 | Intron,Transition Substitution |
| RS2737673 | *NR5A2* | 0.39 (C) | chr. 1 198348333 | Intron,Transition Substitution |
| RS4915195 |  | 0.36 (A) | chr. 1 198417361 | Intergenic/Unknown,Transition Substitution |
| RS4341387 |  | 0.19 (T) | chr. 1 198496069 | Intergenic/Unknown,Transition Substitution |
| RS2066123 |  | 0.38 (C) | chr. 1 198549328 | Intergenic/Unknown,Transversion Substitution |
| RS2790138 |  | 0.15 (C) | chr. 1 198596633 | Intergenic/Unknown,Transition Substitution |
| RS12117955 |  | 0.59 (C) | chr. 1 198692056 | Intergenic/Unknown,Transversion Substitution |
| RS12120084 | *KIF14* | 0.41 (C) | chr. 1 198789189 | Mis-sense Mutation,Transversion Substitution |
| RS6665951 | *KIF14* | 0.21 (T) | chr. 1 198824986 | Silent Mutation,Transition Substitution |
| RS12126676 | *DDX59* | 0.48 (C) | chr. 1 198867443 | Intron,Transition Substitution |
| RS6665604 | *DDX59* | 0.05 (T) | chr. 1 198884861 | Silent Mutation,Transition Substitution |
| RS7544548 |  | 0.49 (T) | chr. 1 198955923 | Intergenic/Unknown,Transition Substitution |
| RS6427859 | *CAMSAP1L1* | 0.22 (A) | chr. 1 199046776 | Intron,Transversion Substitution |
| RS2292099 |  | 0.08 (G) | chr. 1 199110963 | Intergenic/Unknown,Transversion Substitution |
| RS296533 | *C1orf106* | 0.39 (T) | chr. 1 199132391 | Intron,Transversion Substitution |
| RS2275485 | *KIF21B* | 0.14 (G) | chr. 1 199222911 | Silent Mutation,Transition Substitution |
| RS2297909 | *KIF21B* | 0.35 (A) | chr. 1 199226930 | Intron,Transition Substitution |
| RS6692235 | *KIF21B* | 0.90 (G) | chr. 1 199236389 | Intron,Transition Substitution |
| RS957957 | *KIF21B* | 0.48 (A) | chr. 1 199254421 | Intron,Transition Substitution |
| RS3850625 | *CACNA1S* | 0.08 (A) | chr. 1 199282919 | Mis-sense Mutation,Transition Substitution |
| RS7415038 | *CACNA1S* | 0.46 (G) | chr. 1 199305310 | Silent Mutation,Transition Substitution |
| RS16847745 | *CACNA1S* | 0.11 (C) | chr. 1 199351783 | Intron,Transition Substitution |
| RS831768 |  | 0.07 (T) | chr. 1 199415656 | Intergenic/Unknown,Transition Substitution |
| RS3738270 | *DKFZp434B1231* | 0.31 (C) | chr. 1 199461742 | Mis-sense Mutation,Transition Substitution |
| RS4915502 |  | 0.11 (C) | chr. 1 199506619 | Intergenic/Unknown,Transition Substitution |
