# Supplementary material for: Genetic and functional association of FAM5C with myocardial infarction
Source: BMC Med Genet. 2008 Apr 22;9:33. doi: 10.1186/1471-2350-9-33 (PMC2383879; doi:10.1186/1471-2350-9-33)
Supplement: Additional file 4 — Table 2. A list of primer sets used to amplify genomic regions of FAM5C for sequencing. [file 1471-2350-9-33-S4.doc]

| Additional files, Table 2 | | | |
| --- | --- | --- | --- |
| Primers sets for each *FAM5C* intron 7region are as follows (listed 5'-3') | | | |
| Region | Set | Primer Information | |
| 1 | 1 | Forward (1FA) | TCTGTCCTCTCCTGTTTTTGC |
|  |  | Reverse (1RA) | CCCAAGAAGCTGAAATCCAA |
| 2 | 1 | Forward (2FA) | TGATCCTTTTGAAACCCTCAA |
|  |  | Reverse (2RA) | CCACATTGCACAGATTTGGA |
| 2 | 2 | Forward (2FB) | CAAAGGTCCCTTATTCCAACC |
|  |  | Reverse (2RB) | TCTGGAGAGACAGTGACAGAATTT |
| 3 | 1 | Forward (3FA) | TGTTTCCTAAAGCTGCGT |
|  |  | Reverse (3RA) | TTCTTGTGTGTGACATGCCA |
| 3 | 2 | Forward (3FB) | GGCAAGTGGGAATACACATAC |
|  |  | Reverse (3RB) | AGGGTGGAGAGCCAGTT |
| 3 | 3 | Forward (3FC) | AAACACCTGCTGTCCTTTCTG |
|  |  | Reverse (3RC) | TCTGTGGCTTTAATTCTTTCTTCA |
| 4 | 1 | Forward (4FA) | AGGGAGAAGAGTGGTTGTCCT |
|  |  | Reverse (4RA) | TCCAAGGTCCTATCCTGCTTT |
| 4 | 2 | Forward (4FB) | AAAGCAGGATAGGACCTTGGA |
|  |  | Reverse (4RB) | TGCTTTAGAACAGCGTGACC |
| 5 | 1 | Forward (5FA) | AGGGACATCAGAGTGAGAAAGC |
|  |  | Reverse (5RA) | CCAAAAATGTCTCTCCATGC |
| 5 | 2 | Forward (5FB) | GCATGGAGAGACATTTTTGG |
|  |  | Reverse (5RB) | TCTTCACACTAAGCATGCTGC |
| 5 | 3 | Forward (5FC) | GCAGCATGCTTAGTGTGAAGA |
|  |  | Reverse (5RC) | GCATGCAAATAGCATTCTGTG |
| 6 | 1 | Forward (6FA) | GGATACCAAAATTAGCATTCCA |
|  |  | Reverse (6RA) | AACCTATGAAAAACAGGCCTCA |
| 6 | 2 | Forward (6FB) | TGCCAAATTTCACAGGTGCT |
|  |  | Reverse (6RB) | AAAGCCAAGGGATTCTTTCAA |
| 7 | 1 | Forward (7FA) | TGTTTATGGCTAACTTTCCTGCT |
|  |  | Reverse (7RA) | AAATCAAGAGGGTGAAAACTACA |
| 7 | 2 | Forward (7FB) | CCACAGCAAACTGACCTTCA |
|  |  | Reverse (7RB) | AGGCAGCCCACACAAGTTAT |
