# Supplementary material for: Genetic and functional association of FAM5C with myocardial infarction
Source: BMC Med Genet. 2008 Apr 22;9:33. doi: 10.1186/1471-2350-9-33 (PMC2383879; doi:10.1186/1471-2350-9-33)
Supplement: Additional file 5 — Table 3. Sixteen significant SNPs identified in peakwide screen of chromosome 1 ACS linkage peak and replication of three associated SNPs in CATHGEN MI case-control sample. [file 1471-2350-9-33-S5.doc]

| Additional files, Table 3 | | | | | | | | |
| --- | --- | --- | --- | --- | --- | --- | --- | --- |
|  |  |  | GENECARD ACS | | | | CATHGEN MI | |
| SNP | Locus | Chrom1 location (build36) | Twopoint | | APL | | Allele test | |
|  |  |  | LOD (DOM) | LOD (REC) | p-value | variance | p-value | Odds Ratio |
| RS339577 |  | 193,197,164 | 0.12 | 0.03 | 0.002 | 21.21 | 0.221 | 0.78 |
| RS3009350 |  | 192,869,230 | 0.39 | 0.35 | 0.003 | 15.77 | 0.908 | 1.02 |
| RS10921817 |  | 193,452,046 | 0.04 | 0.08 | 0.006 | 10.25 | 0.110 | 1.43 |
| RS10798301 | RC3H1 | 172,215,955 | 0.30 | 0.31 | 0.009 | 15.54 | 0.925 | 1.02 |
| RS7528236 |  | 174,019,909 | 0.47 | 0.52 | 0.013 | 8.85 | 0.590 | 1.12 |
| RS1324713 |  | 193,643,558 | 0.20 | 0.57 | 0.019 | 15.79 | 0.042 | 1.53 |
| RS16841912 | DENND1B | 195,969,099 | 0.06 | 0.05 | 0.019 | 12.29 | 0.199 | 0.76 |
| RS6676084 | ASPM | 195,360,653 | 0.62 | 0.50 | 0.024 | 40.65 | 0.397 | 1.19 |
| RS1891586 | FAM5C | 188,430,617 | 1.54 | 1.34 | 0.027 | 21.84 | 0.008 | 0.57 |
| RS12131585 | TROVE2 | 191,301,636 | 0.20 | 0.52 | 0.028 | *2.86* | 0.323 | 0.69 |
| RS12092963 | RGL1 | 181,938,444 | 1.36 | 1.15 | 0.030 | 6.20 | 0.780 | 1.08 |
| RS1929230 |  | 192,965,181 | 0.39 | 0.32 | 0.031 | 18.29 | 0.859 | 0.96 |
| RS12145507 |  | 188,237,152 | 0.27 | 0.09 | 0.040 | 14.69 | 0.331 | 1.29 |
| RS10912660 | ANKRD45 | 171,904,141 | 2.13 | 2.45 | 0.040 | 32.54 | 0.313 | 1.23 |
| RS2239816 | C1ORF9 | 170,768,266 | 0.13 | 0.14 | 0.043 | 17.09 | 0.041 | 1.56 |
| RS1955245 |  | 186,491,290 | 0.30 | 0.22 | 0.044 | 26.94 | 0.944 | 0.99 |
