# Supplementary material for: Genetic and functional association of FAM5C with myocardial infarction
Source: BMC Med Genet. 2008 Apr 22;9:33. doi: 10.1186/1471-2350-9-33 (PMC2383879; doi:10.1186/1471-2350-9-33)
Supplement: Additional file 6 — Table 4. tagSNP genotyping results for C1orf9 and an intergenic region surrounding rs1324713 in GENECARD ACS families. [file 1471-2350-9-33-S6.doc]

| Additional files, Table 4 | | | | | | | | | | |
| --- | --- | --- | --- | --- | --- | --- | --- | --- | --- | --- |
| SNP | Locus | Chrom1 location (build36) | Twopoint | | APL | | PDT | | GenoPDT | |
|  |  |  | LOD (DOM) | LOD (REC) | p-value | variance | p-value | chisquare | p-value | chisquare |
| RS2239816 | C1ORF9 | 170,768,266 | 0.158 | 0.172 | **0.050** | 15.919 | **0.024** | 5.121 | **0.009** | 9.345 |
| RS6695152 | C1ORF9 | 170,774,335 | 0.003 | 0.033 | 0.583 | 17.249 | 0.547 | 0.364 | 0.262 | 2.681 |
| RS12758548 | C1ORF9 | 170,785,519 | 0.574 | 0.755 | 0.283 | 14.222 | 0.564 | 0.333 | 0.442 | 1.633 |
| RS2516064 | C1ORF9 | 170,789,514 | 0.512 | **1.012** | 0.468 | 14.793 | 0.170 | 1.882 | 0.309 | 2.350 |
| RS10752994 | C1ORF9 | 170,821,224 | 0.241 | 0.300 | 0.763 | 20.372 | 0.686 | 0.164 | 3.935 | 3.935 |
| RS1053381 | C1ORF9 | 170,847,160 | 0.487 | 0.737 | 0.276 | 6.232 | 0.157 | 2.000 | 0.157 | 2.000 |
| RS3850641 | TNFSF4 | 171,442,455 | **1.288** | **1.293** | 0.364 | 9.659 | 0.180 | 1.800 | 0.480 | 1.467 |
| RS12125032 |  | 193,608,350 | 0.229 | 0.265 | 0.905 | 33.198 | 0.317 | 1.000 | 0.575 | 1.109 |
| RS17668176 |  | 193,610,411 | 0.758 | **1.090** | 0.791 | 30.091 | 0.439 | 0.600 | 0.297 | 2.431 |
| RS12729977 |  | 193,615,561 | 0.482 | 0.891 | 0.524 | 20.917 | 1.000 | 0.000 | 0.444 | 1.625 |
| RS12723502 |  | 193,628,999 | 0.429 | 0.248 | 0.362 | 16.634 | 0.160 | 1.976 | 0.116 | 4.301 |
| RS6428283 |  | 193,630,035 | 0.359 | 0.452 | 0.449 | 22.146 | 1.000 | 0.000 | 0.916 | 0.176 |
| RS1324713 |  | 193,643,558 | 0.310 | 0.799 | **0.005** | 13.705 | 0.139 | 2.189 | 0.174 | 3.498 |
| RS12141389 |  | 193,649,088 | 0.087 | 0.095 | 0.365 | 21.789 | 0.139 | 2.189 | 0.265 | 2.658 |
| RS16838352 |  | 193,657,269 | 0.000 | 0.000 | 0.447 | 16.944 | 0.131 | 2.286 | 0.255 | 2.734 |
| RS12401422 |  | 193,660,147 | 0.029 | 0.156 | 0.161 | 11.304 | 0.160 | 1.976 | 0.316 | 2.307 |
| RS7523327 |  | 193,660,236 | 0.102 | 0.140 | 0.474 | 24.117 | 0.069 | 3.314 | 0.145 | 3.860 |
| RS12403940 |  | 193,682,727 | 0.017 | 0.000 | 0.353 | 21.248 | **0.040** | 4.235 | **0.046** | 6.148 |
| RS12128390 |  | 193,686,693 | 0.000 | 0.000 | 0.758 | 7.330 | 0.527 | 0.400 | 0.683 | 0.762 |
| RS12139521 |  | 193,687,149 | 0.165 | 0.349 | 0.432 | 22.097 | 0.258 | 1.280 | 0.373 | 1.970 |
