# Supplementary material for: Genetic and functional association of FAM5C with myocardial infarction
Source: BMC Med Genet. 2008 Apr 22;9:33. doi: 10.1186/1471-2350-9-33 (PMC2383879; doi:10.1186/1471-2350-9-33)
Supplement: Additional file 7 — Table 5. tagSNP genotyping results for FAM5C in GENECARD ACS families. [file 1471-2350-9-33-S7.doc]

| Additional files, Table 5 | | | | | | | | | | | | | | |
| --- | --- | --- | --- | --- | --- | --- | --- | --- | --- | --- | --- | --- | --- | --- |
| FAM5C tagSNPs | | | | | | | | | | | | | | |
| SNP | Locus | Chrom1 location (build36) | Minor Allele Frequency | Twopoint | | APL | | | PDT | | | GenoPDT | | |
|  |  |  |  | LOD (DOM) | LOD (REC) | p-value | -log10 (p-value) | variance | p-value | -log10 (p-value) | chi square | p-value | -log10 (p-value) | chi square |
| RS10920501 | FAM5C | 188,328,568 | 0.2 | 0.8 | 1.0 | 0.455 | 0.342 | 13.5 | 0.516 | 0.287 | 0.421 | 0.516 | 0.287 | 0.421 |
| RS12125036 | FAM5C | 188,332,063 | 0.25 | 0.4 | 0.3 | 0.507 | 0.295 | 6.6 | 0.763 | 0.117 | 0.091 | 0.763 | 0.117 | 0.091 |
| RS1935881 | FAM5C | 188,333,009 | 0.386 | 0.5 | 0.4 | 0.394 | 0.405 | 27.7 | 0.131 | 0.883 | 2.283 | 0.190 | 0.722 | 3.324 |
| RS12142564 | FAM5C | 188,338,626 | 0.092 | 0.9 | **1.3** | 0.767 | 0.115 | 12.0 | 0.853 | 0.069 | 0.034 | 0.853 | 0.069 | 0.034 |
| RS17375171 | FAM5C | 188,339,790 | 0.108 | 0.3 | 0.3 | 0.105 | 0.980 | 19.6 | 0.285 | 0.545 | 1.143 | 0.285 | 0.545 | 1.143 |
| RS7412309 | FAM5C | 188,344,664 | 0.37 | 0.3 | 0.2 | 0.970 | 0.0 | 12.8 | 0.132 | 0.9 | 2.27 | 0.132 | 0.9 | 2.27 |
| RS2185836 | FAM5C | 188,349,217 | 0.34 | 1.0 | **1.1** | 0.346 | 0.5 | 27.4 | 0.063 | 1.2 | 3.45 | 0.268 | 0.6 | 2.64 |
| RS10800905 | FAM5C | 188,363,448 | 0.11 | 0.8 | 0.1 | 0.712 | 0.1 | 8.7 | 0.273 | 0.6 | 1.20 | 0.450 | 0.3 | 1.60 |
| RS2419370 | FAM5C | 188,375,504 | 0.49 | 0.6 | 0.7 | 0.339 | 0.5 | 38.5 | **0.022** | 1.7 | 5.26 | 0.052 | 1.3 | 5.93 |
| RS4576663 | FAM5C | 188,389,818 | 0.40 | 0.0 | 0.0 | 0.956 | 0.0 | 6.3 | 1.000 | 0.0 | 0.00 | 1.000 | 0.0 | 0.00 |
| RS10920653 | FAM5C | 188,390,532 | 0.13 | 0.1 | 0.1 | 0.672 | 0.2 | 25.7 | 0.862 | 0.1 | 0.03 | 0.709 | 0.1 | 0.69 |
| RS10047244 | FAM5C | 188,395,924 | 0.11 | 0.2 | 0.2 | 0.780 | 0.1 | 22.6 | 0.862 | 0.1 | 0.03 | 0.709 | 0.1 | 0.69 |
| RS12076854 | FAM5C | 188,401,694 | 0.35 | 0.2 | 0.2 | 0.700 | 0.2 | 12.6 | 0.317 | 0.5 | 1.00 | 0.317 | 0.5 | 1.00 |
| RS1093086 | FAM5C | 188,402,434 | 0.23 | 0.3 | 0.0 | 0.274 | 0.6 | 15.9 | 0.063 | 1.2 | 3.46 | 0.188 | 0.7 | 3.34 |
| RS815331 | FAM5C | 188,406,170 | 0.11 | 0.2 | 0.4 | 0.158 | 0.8 | 7.0 | 0.157 | 0.8 | 2.00 | 0.157 | 0.8 | 2.00 |
| RS2990996 | FAM5C | 188,411,850 | 0.49 | 0.3 | 0.4 | 0.073 | 1.1 | 21.2 | **0.018** | 1.7 | 5.57 | 0.063 | 1.2 | 5.52 |
| RS815343 | FAM5C | 188,422,256 | 0.35 | **1.7** | **1.3** | **0.045** | 1.3 | 22.3 | **0.023** | 1.6 | 5.16 | 0.081 | 1.1 | 5.03 |
| RS1891586 | FAM5C | 188,430,617 | 0.11 | **1.5** | **1.3** | **0.027** | 1.6 | 21.8 | **0.028** | 1.6 | 4.84 | 0.070 | 1.2 | 5.31 |
| RS9427746 | FAM5C | 188,438,263 | 0.43 | 0.1 | 0.2 | 0.097 | 1.0 | 23.1 | **0.033** | 1.5 | 4.55 | 0.175 | 0.8 | 3.48 |
| RS4399162 | FAM5C | 188,468,347 | 0.17 | 0.1 | 0.0 | 0.401 | 0.4 | 23.7 | 0.132 | 0.9 | 2.27 | 0.132 | 0.9 | 2.27 |
| RS17368045 | FAM5C | 188,480,155 | 0.19 | 0.0 | 0.0 | 0.875 | 0.1 | 18.2 | 0.695 | 0.2 | 0.15 | 0.073 | 1.1 | 5.25 |
| RS1855241 | FAM5C | 188,498,748 | 0.33 | 0.6 | 0.5 | 0.135 | 0.9 | 27.9 | 0.056 | 1.3 | 3.67 | 0.150 | 0.8 | 3.80 |
| RS10920678 | FAM5C | 188,506,530 | 0.40 | 0.5 | 0.4 | **0.050** | 1.3 | 25.0 | 0.077 | 1.1 | 3.13 | 0.143 | 0.8 | 3.89 |
| RS480692 | FAM5C | 188,526,284 | 0.10 | 0.1 | 0.1 | 0.208 | 0.7 | 25.7 | **0.022** | 1.7 | 5.23 | **0.035** | 1.5 | 6.70 |
| RS510498 | FAM5C | 188,534,730 | 0.07 | 0.1 | 0.1 | 0.662 | 0.2 | 12.6 | 1.000 | 0.0 | 0.00 | 0.664 | 0.2 | 0.82 |
| RS576503 | FAM5C | 188,538,657 | 0.48 | 0.4 | 0.4 | 0.075 | 1.1 | 26.3 | 0.056 | 1.3 | 3.67 | 0.150 | 0.8 | 3.80 |
| RS12724000 | FAM5C | 188,618,300 | 0.15 | **1.3** | **1.2** | 0.316 | 0.5 | 7.5 | 0.090 | 1.0 | 2.88 | 0.208 | 0.7 | 3.14 |
| RS7545105 | FAM5C | 188,632,263 | 0.13 | 0.4 | 0.3 | 0.993 | 0.0 | 11.1 | 0.670 | 0.2 | 0.18 | 0.705 | 0.2 | 0.70 |
| RS1171150 | FAM5C | 188,633,119 | 0.49 | 0.2 | 0.3 | 0.870 | 0.1 | 15.5 | 0.493 | 0.3 | 0.47 | 0.778 | 0.1 | 0.50 |
| RS1171148 | FAM5C | 188,644,611 | 0.49 | 0.3 | 0.2 | 0.472 | 0.3 | 24.5 | 0.096 | 1.0 | 2.77 | 0.358 | 0.4 | 2.06 |
| RS1171046 | FAM5C | 188,649,658 | 0.46 | 0.0 | 0.0 | 0.490 | 0.3 | 9.8 | 0.827 | 0.1 | 0.05 | 0.558 | 0.3 | 1.17 |
| RS1171043 | FAM5C | 188,652,173 | 0.44 | 0.2 | 0.2 | 0.472 | 0.3 | 22.4 | 0.204 | 0.7 | 1.61 | 0.474 | 0.3 | 1.49 |
| RS16832316 | FAM5C | 188,656,920 | 0.10 | 0.0 | 0.0 | 0.293 | 0.5 | 15.2 | 0.842 | 0.1 | 0.04 | 0.973 | 0.0 | 0.06 |
| RS1171041 | FAM5C | 188,658,454 | 0.33 | 0.2 | 0.4 | 0.666 | 0.2 | 20.5 | 0.639 | 0.2 | 0.22 | 0.883 | 0.1 | 0.25 |
| RS872177 | FAM5C | 188,659,068 | 0.26 | **1.0** | 0.8 | 0.313 | 0.5 | 19.4 | 0.433 | 0.4 | 0.62 | 0.660 | 0.2 | 0.83 |
| RS2134098 | FAM5C | 188,660,894 | 0.24 | 0.7 | **1.1** | 0.370 | 0.4 | 11.3 | 0.513 | 0.3 | 0.43 | 0.513 | 0.3 | 0.43 |
| RS1171037 | FAM5C | 188,677,302 | 0.40 | 0.3 | 0.4 | 0.792 | 0.1 | 17.6 | 0.858 | 0.1 | 0.03 | 0.953 | 0.0 | 0.10 |
| RS1171023 | FAM5C | 188,685,276 | 0.44 | 0.0 | 0.2 | 0.375 | 0.4 | 26.6 | 0.866 | 0.1 | 0.03 | 0.649 | 0.2 | 0.86 |
| RS1171024 | FAM5C | 188,685,584 | 0.44 | 0.0 | 0.1 | 0.294 | 0.5 | 18.4 | 0.083 | 1.1 | 3.00 | 0.092 | 1.0 | 4.77 |
| RS10920722 | FAM5C | 188,694,717 | 0.09 | **3.2** | **3.3** | 0.172 | 0.8 | 9.6 | 0.317 | 0.5 | 1.00 | 0.487 | 0.3 | 1.44 |
| RS10920725 | FAM5C | 188,707,160 | 0.17 | **3.4** | **3.5** | 0.160 | 0.8 | 11.7 | 0.317 | 0.5 | 1.00 | 0.438 | 0.4 | 1.65 |
| RS12121097 | FAM5C | 188,708,104 | 0.10 | 0.0 | 0.0 | 0.262 | 0.6 | 14.3 | 0.144 | 0.8 | 2.13 | 0.274 | 0.6 | 2.59 |
| RS2490271 | FAM5C | 188,709,594 | 0.11 | 0.0 | 0.0 | 0.244 | 0.6 | 10.3 | 0.513 | 0.3 | 0.43 | 0.326 | 0.5 | 2.24 |
| RS1697593 | FAM5C | 188,720,295 | 0.43 | 0.9 | 0.8 | 0.363 | 0.4 | 29.4 | 0.758 | 0.1 | 0.10 | 0.854 | 0.1 | 0.32 |
| RS814925 | FAM5C | 188,722,485 | 0.06 | 0.2 | 0.3 | 0.465 | 0.3 | 26.8 | 0.869 | 0.1 | 0.03 | 0.734 | 0.1 | 0.62 |
| RS11581737 |  | 188,726,896 | 0.17 | **2.2** | **2.3** | 0.464 | 0.3 | 12.5 | 0.549 | 0.3 | 0.36 | 0.752 | 0.1 | 0.57 |
| RS703934 |  | 188,729,913 | 0.16 | 0.1 | 0.4 | 0.805 | 0.1 | 22.0 | 0.876 | 0.1 | 0.02 | 0.752 | 0.1 | 0.57 |
